# Differential diagnosis of perinatal hypophosphatasia: radiologic perspectives

**DOI:** 10.1007/s00247-018-4239-0

**Published:** 2018-10-03

**Authors:** Amaka C. Offiah, Jerry Vockley, Craig F. Munns, Jun Murotsuki

**Affiliations:** 1 Academic Unit of Child Health, Sheffield Children’s NHS Foundation Trust, University of Sheffield, Western Bank, Sheffield, S10 2TH UK; 2 School of Medicine and Children’s Hospital of Pittsburgh, University of Pittsburgh, Pittsburgh, PA USA; 3 The Children’s Hospital at Westmead, Westmead, NSW Australia; 4 Sydney Medical School, The University of Sydney, University of Sydney NSW, Sydney, Australia; 5 Aoba Ward, Miyagi Children’s Hospital, Sendai, Miyagi Prefecture Japan

**Keywords:** Computed tomography, Hypophosphatasia, Metabolic bone disease, Perinatal, Radiography, Skeletal dysplasia, Ultrasound

## Abstract

Perinatal hypophosphatasia (HPP) is a rare, potentially life-threatening, inherited, systemic metabolic bone disease that can be difficult to recognize in utero and postnatally. Diagnosis is challenging because of the large number of skeletal dysplasias with overlapping clinical features. This review focuses on the role of fetal and neonatal imaging modalities in the differential diagnosis of perinatal HPP from other skeletal dysplasias (e.g., osteogenesis imperfecta, campomelic dysplasia, achondrogenesis subtypes, hypochondrogenesis, cleidocranial dysplasia). Perinatal HPP is associated with a broad spectrum of imaging findings that are characteristic of but do not occur in all cases of HPP and are not unique to HPP, such as shortening, bowing and angulation of the long bones, and slender, poorly ossified ribs and metaphyseal lucencies. Conversely, absent ossification of whole bones is characteristic of severe lethal HPP and is associated with very few other conditions. Certain features may help distinguish HPP from other skeletal dysplasias, such as sites of angulation of long bones, patterns of hypomineralization, and metaphyseal characteristics. In utero recognition of HPP allows for the assembly and preparation of a multidisciplinary care team before delivery and provides additional time to devise treatment strategies.

## Introduction

Hypophosphatasia (HPP) is a rare, inherited, systemic, metabolic bone disease caused by low tissue-nonspecific alkaline phosphatase activity [1–3]. In patients with HPP, low alkaline phosphatase activity results in the accumulation of phosphorylated substrates, specifically inorganic pyrophosphate, pyridoxal 5′-phosphate and phosphoethanolamine [1, 3–5]. Elevated inorganic pyrophosphate levels inhibit mineralization of the bone matrix, leading to hypomineralization of the skeleton [2, 6–8]. The inability of pyridoxal 5′-phosphate, the circulating form of vitamin B6, to cross the blood-brain barrier likely contributes to the seizures observed in some infants with HPP [2, 8].

HPP is a clinically heterogeneous disease traditionally categorized by the age of onset of the first signs and symptoms as perinatal onset (in utero and at birth), infantile onset (age < 6 months), childhood onset (age ≥ 6 months to <18 years), and adult onset (age ≥ 18 years) or, in patients with only dental manifestations, as odonto-HPP [2, 3, 9]. Characteristic signs, symptoms and complications of perinatal HPP include skeletal manifestations (e.g., hypomineralization, chest deformity, bowing, craniosynostosis) [9, 10], vitamin B–responsive seizures [9, 11–13] and respiratory failure [11, 14]. Before the availability of enzyme replacement therapy, mortality among patients with perinatal/infantile HPP was high, ranging from 58% to 100% within the first year of life [15–17]. The incidence of HPP has been estimated to be 1:100,000 in Ontario, Canada, based on the local birth rate in 1957 [9]. The prevalence of perinatal and infantile HPP in Europe has been estimated to be 1:538,000, based on molecular diagnoses made from 2000 to 2009 [18]. Local populations with a higher incidence of HPP include the Mennonite communities in Canada [19] and an endogamous village in Hungary [20, 21]. Because of the rarity of HPP, its true incidence and prevalence remain unknown [3].

HPP is confirmed with consistently low age- and gender-adjusted alkaline phosphatase activity in conjunction with medical history and physical findings, radiologic findings, elevated levels of tissue-nonspecific alkaline phosphatase substrates or sequencing of the *ALPL* gene [3, 22, 23]. In utero and postnatal recognition and diagnosis of perinatal HPP based on radiologic findings can be challenging because of features that overlap with many of the more than 400 other skeletal dysplasias, the phenotypic variability and a lack of information about the in utero natural history of HPP [24–26]. The skeletal abnormalities and the gestational and postnatal ages at which they manifest vary across skeletal dysplasias, including HPP. Many sonographic and radiographic findings are not pathognomonic for a specific disorder, as obtaining reliable information regarding skeletal mineralization is difficult with prenatal sonography and computed tomography (CT). These difficulties are confounded by a general lack of familiarity with HPP among the health care providers who perform prenatal ultrasound (US) and neonatal imaging. In addition, abnormalities can be detected at earlier gestational ages than they have in the past [27], underscoring the need for obstetricians, ultrasonographers and radiologists to possess in-depth knowledge of the appearance of the fetal skeleton at all gestational ages [28]. This review focuses on the role of fetal and neonatal imaging modalities in the differential diagnosis of perinatal HPP.

### Prenatal imaging

Prenatal diagnosis of skeletal dysplasia relies on cross-sectional imaging modalities (US, CT and magnetic resonance imaging [MRI]), whereas postnatal diagnosis relies more heavily on radiography [29]. The International Society of Ultrasound in Obstetrics and Gynecology [30] and the United Kingdom’s National Institute for Health and Care Excellence [31] recommend that all pregnant women undergo US scanning at 10 to 14 weeks to establish gestational age and at 18 to 22 weeks to screen for structural anomalies. Thereafter, the frequency of fetal monitoring depends on the severity of findings, the mother’s health and the family’s wishes. High-resolution US is required to clearly identify the skeletal abnormalities of HPP. Two- or three-dimensional (2-D or 3-D) US may be used to visualize the skeleton by gestational week 12 [29]. Although radiologists are usually trained with 2-D images and generally prefer 2-D to 3-D US when reviewing image slices, 3-D US may allow the radiologist to more clearly visualize characteristic dysmorphic findings of the face, hands, feet, vertebrae, ribs and skull sutures in skeletal dysplasias [32].

Prenatal CT is a useful modality when skeletal dysplasia is suspected after sonographic examination [33, 34] and is best performed from 30 weeks’ gestation; image quality is poor at earlier gestational ages because of relatively poor skeletal mineralization and artefacts caused by fetal movement [29, 34]. Although the added diagnostic value of CT over US has not been formally assessed in a large study, CT allows detailed visualization of the fetal skeleton and is less dependent on amniotic fluid volume and fetal position than US [33–35]. The risk associated with fetal radiation is a common concern with prenatal CT; however, the risk to benefit ratio can be relatively low if the radiation dose is kept to a minimum by selecting appropriate technical parameters [34, 36–39]. A recommended threshold of radiation that would have negligible risk to the fetus is 50 milligray [38]. Advances in model-based iterative reconstruction methods for ultra-low-dose fetal CT yield fetal radiation exposures as low as 0.5 milligray while maintaining excellent image quality for the diagnosis of skeletal dysplasias [40].

MRI has shown only limited utility in prenatal diagnosis of skeletal dysplasias and is not routinely used in HPP diagnosis [29, 41]; however, fetal MRI can provide valuable details when targeted US is unable to clarify the diagnosis [42, 43]. Fetal “black bone” MRI, compared with standard MRI sequences, may improve visualization of the mineralized skeleton [44].

### Postnatal imaging

**Fig. 1:**
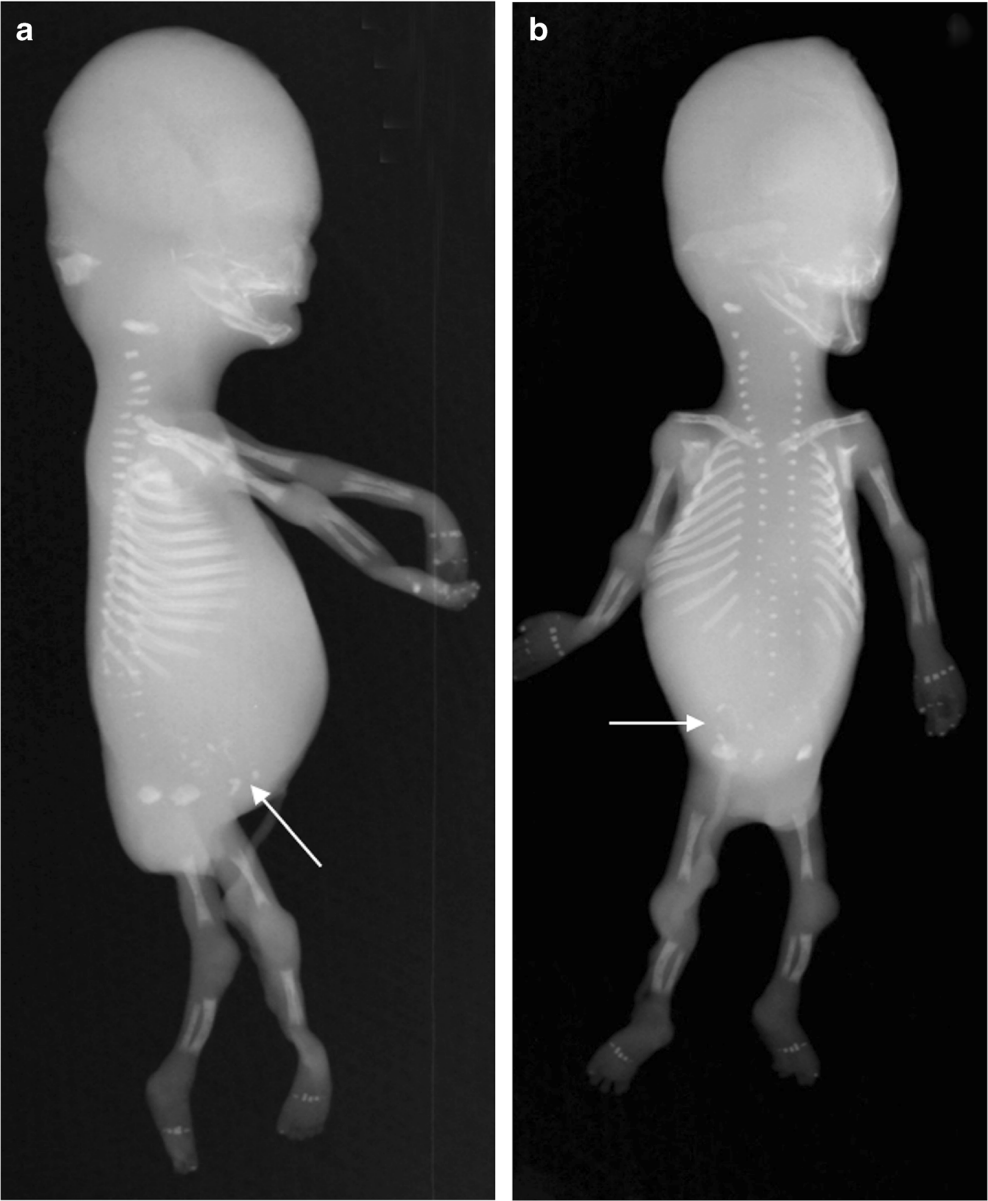
Normal ossification of the fetal skeleton of a male fetus at 11 weeks’ gestation. **a,b** Radiographs in lateral (**a**) and anteroposterior (**b**) projections show absent ossification of skull vault, cervical, thoracic and sacral vertebral bodies, and ischia and pubic bones. This is normal for the gestational age (the pelvic calcification [*arrows*] is probably within the bowel)

**Fig. 2:**
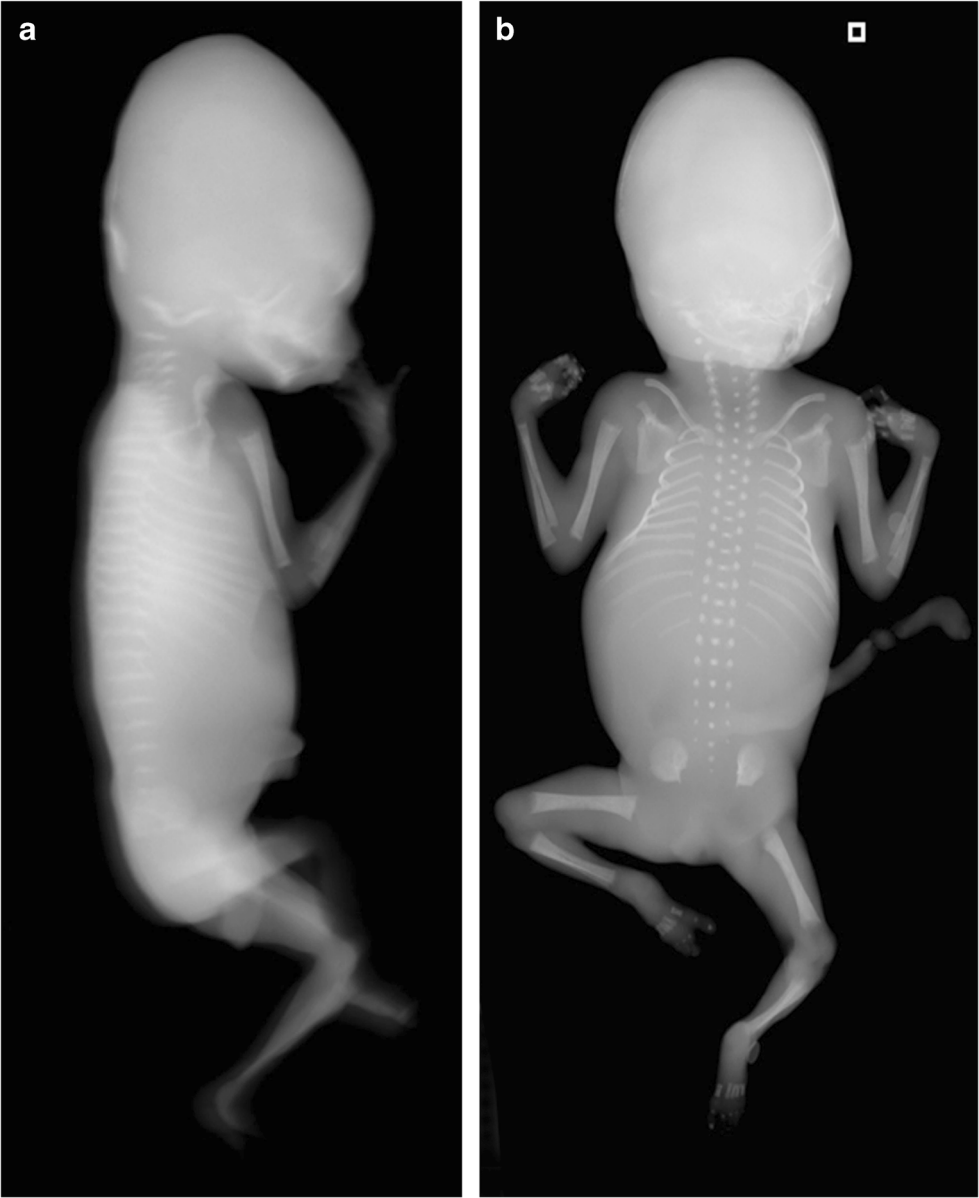
Normal ossification of the fetal skeleton of a male fetus at 14 weeks’ gestation. **a,b** Radiographs in lateral (**a**) and anteroposterior (**b**) projections show ossification of all vertebral bodies, which is in contrast with characteristics shown in the 11-week fetus in Fig. 1; however, there remains absent ossification of the skull vault, much of the skull base, and the ischia and pubic bones

**Fig. 3:**
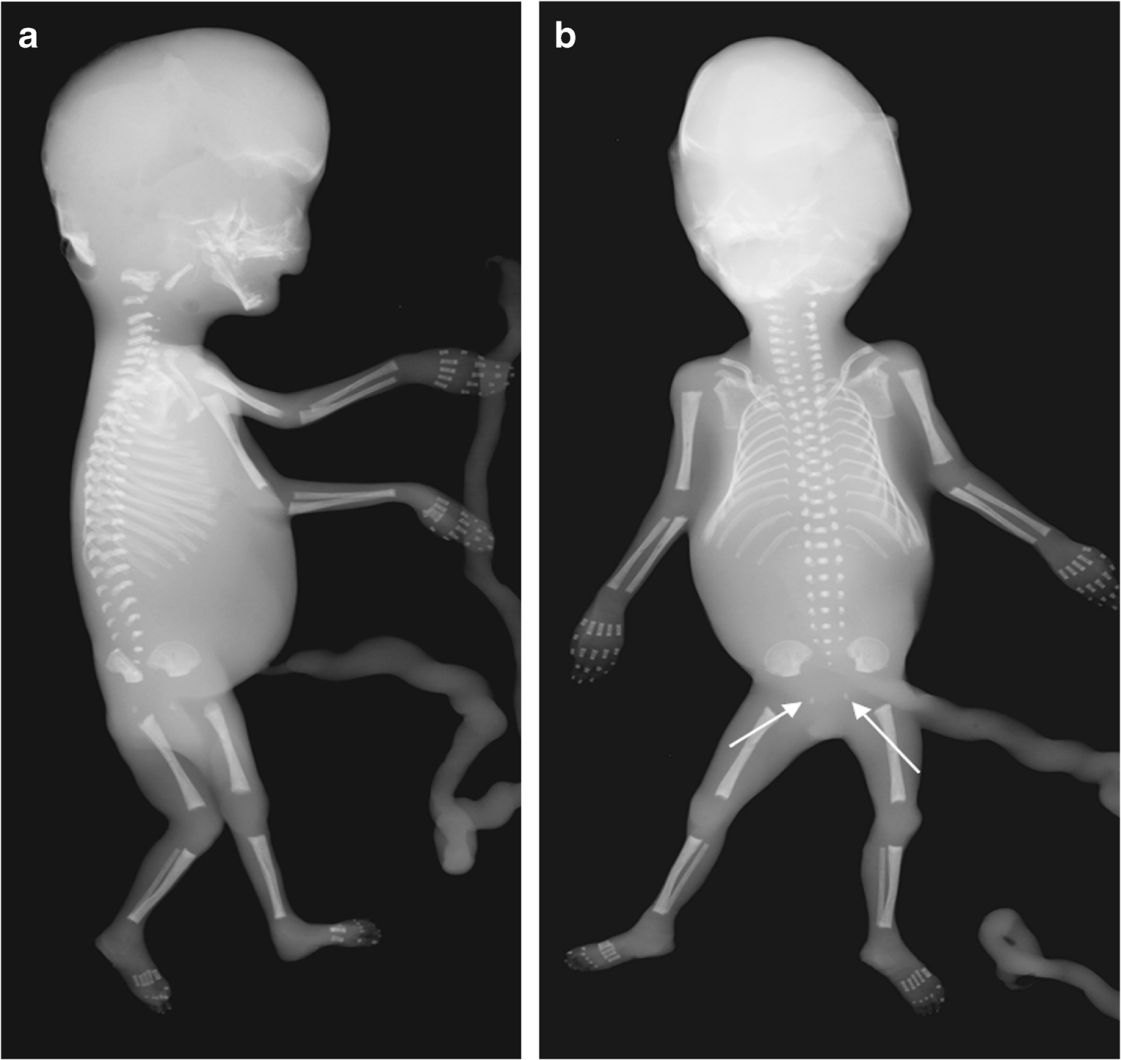
Normal ossification of the fetal skeleton of a male fetus at 15 weeks’ gestation. **a,b** Radiographs in lateral (**a**) and anteroposterior (**b**) projections show early ossification of the ischia at 15 weeks (*arrows*)

A whole-body radiograph of an infant (i.e. babygram) is required for any live-born infant, preterm fetus or stillborn with a suspected constitutional disorder of bone [29, 45]. The babygram includes anteroposterior (AP) and lateral radiographs of the full body length. Figures 1, 2 and 3 show postmortem whole-body radiographs of fetuses with normal skeletons at 11, 14 and 15 weeks’ gestation. In cases of stillbirths, babygrams may be performed using cabinet X-ray machines that visualize all bones of the skeleton on a single projection [28, 29]. For live-born, larger infants, a standard skeletal survey may be required to enhance diagnostic accuracy [29, 45]. The series of radiographs obtained for a standard skeletal survey may vary among institutions but should include the following views: AP and lateral skull, AP chest (including AP thoracic spine), lateral thoracolumbar spine, AP pelvis (including AP lumbar spine), AP one upper limb, AP one lower limb and dorsipalmar left hand [29, 45]. A review of family members’ previous radiographs, if available, may help if any first-degree relatives are suspected of being affected [29].

Cross-sectional imaging modalities (e.g., US, CT, MRI) are generally reserved for specific skeletal and systemic abnormalities. Whole-body MRI findings associated with HPP have been described in children [46] but may be less useful/practical in a perinatal setting.

### Perinatal hypophosphatasia

**Fig. 4:**
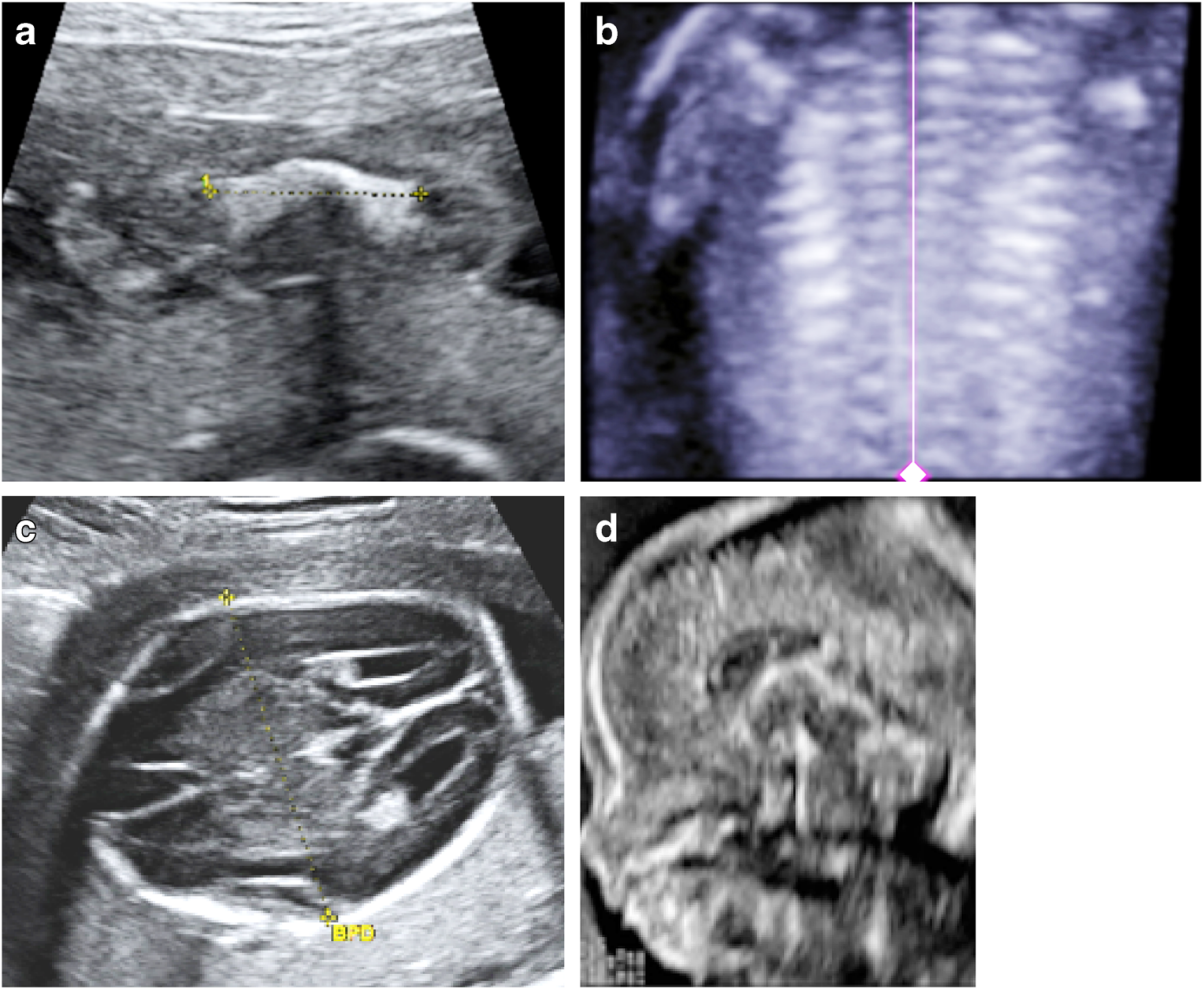
Imaging features of hypophosphatasia of a fetus of unknown gender at 18 weeks’ gestation*.***a-d** Prenatal US scan in a longitudinal view of the femur (**a**) shows a short and angulated femur and a coronal view of the thorax (**b**) shows short irregular ribs. The vertical line highlights the original software calipers used by the sonographer to document thoracic height. Axial (**c**) and sagittal (**d**) views of the skull show a severely underossified cranium. Calipers on the axial view are measuring the biparietal diameter. [Images reproduced with permission from Radcliffe Publishing [38], page 370, case 1, images 1a, 1c, 1f, and 1g]

**Fig. 5:**
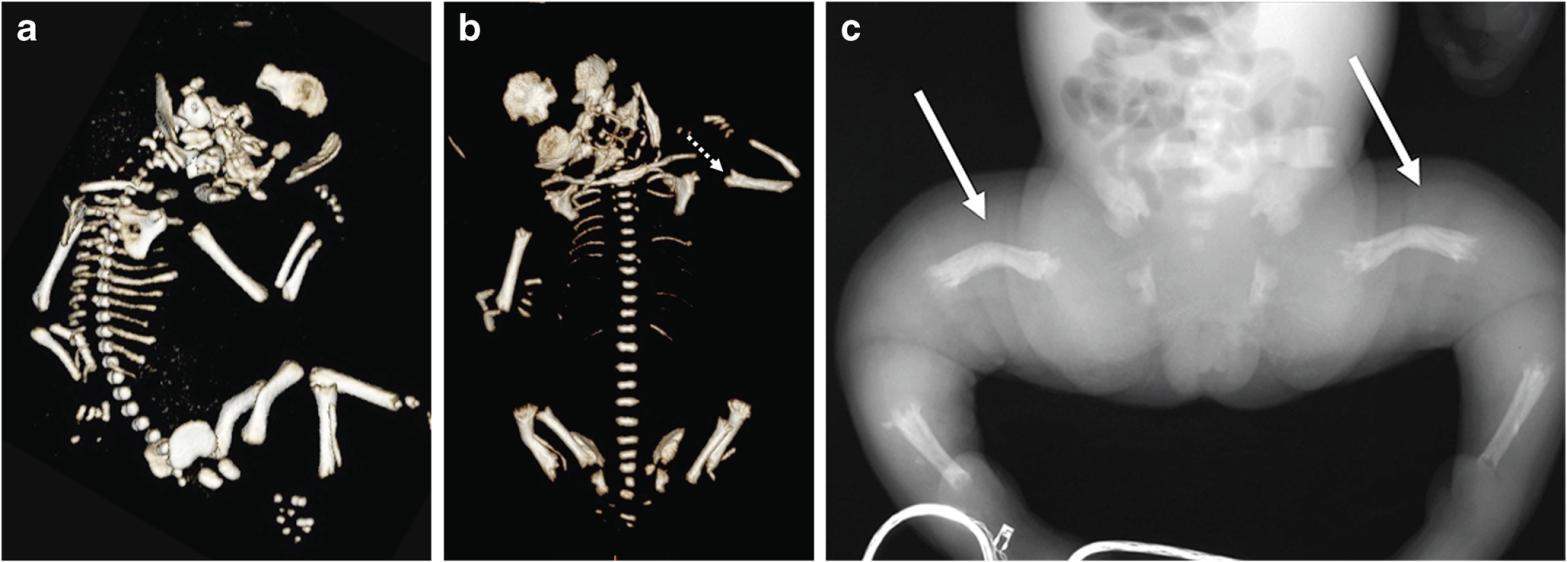
Imaging features of hypophosphatasia in a fetus at 25, 34, and 38 weeks’ gestation*.* The fetus died. **a,b** Three-dimensional reconstructed fetal CT images show deficient ossification of ribs, which is progressive between 25 weeks’ (**a**) and 34 weeks’ (**b**) gestation, and wide cranial sutures and anterior fontanelle. Metaphyseal “tongues” of radiolucency are best appreciated on the CT scan at 34 weeks (*dashed arrow*). **c** Postmortem anteroposterior radiograph of the same fetus at 38 weeks’ gestation shows bowed femora (*arrows*) and absent ossification of pedicles

**Fig. 6:**
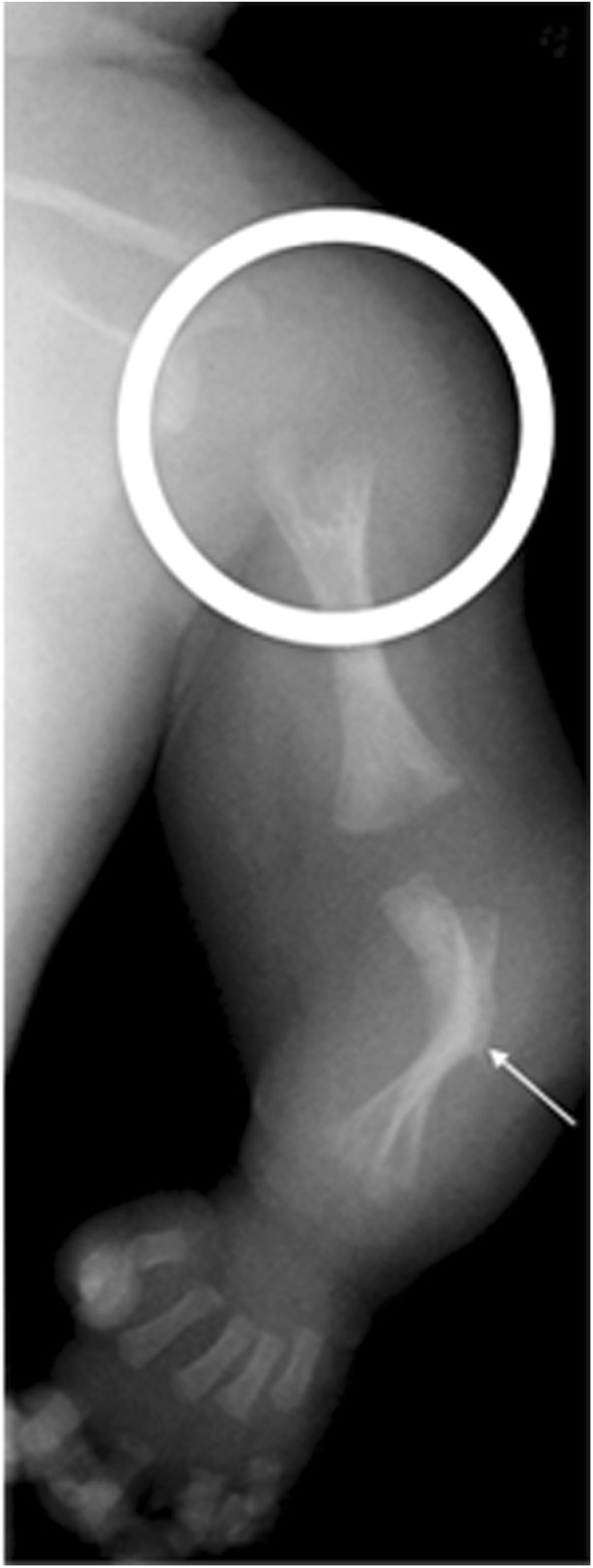
Imaging features of hypophosphatasia of an infant of unknown gender at birth. Anteroposterior radiograph of the left upper limb shows metaphyseal “tongues” of radiolucency of the left proximal humerus (*circled*), with bowing and spurring (*arrow*) of the radius and ulna. [Image reproduced with permission from Radcliffe Publishing [38], page 374, case 11, image 11c]

**Fig. 7:**
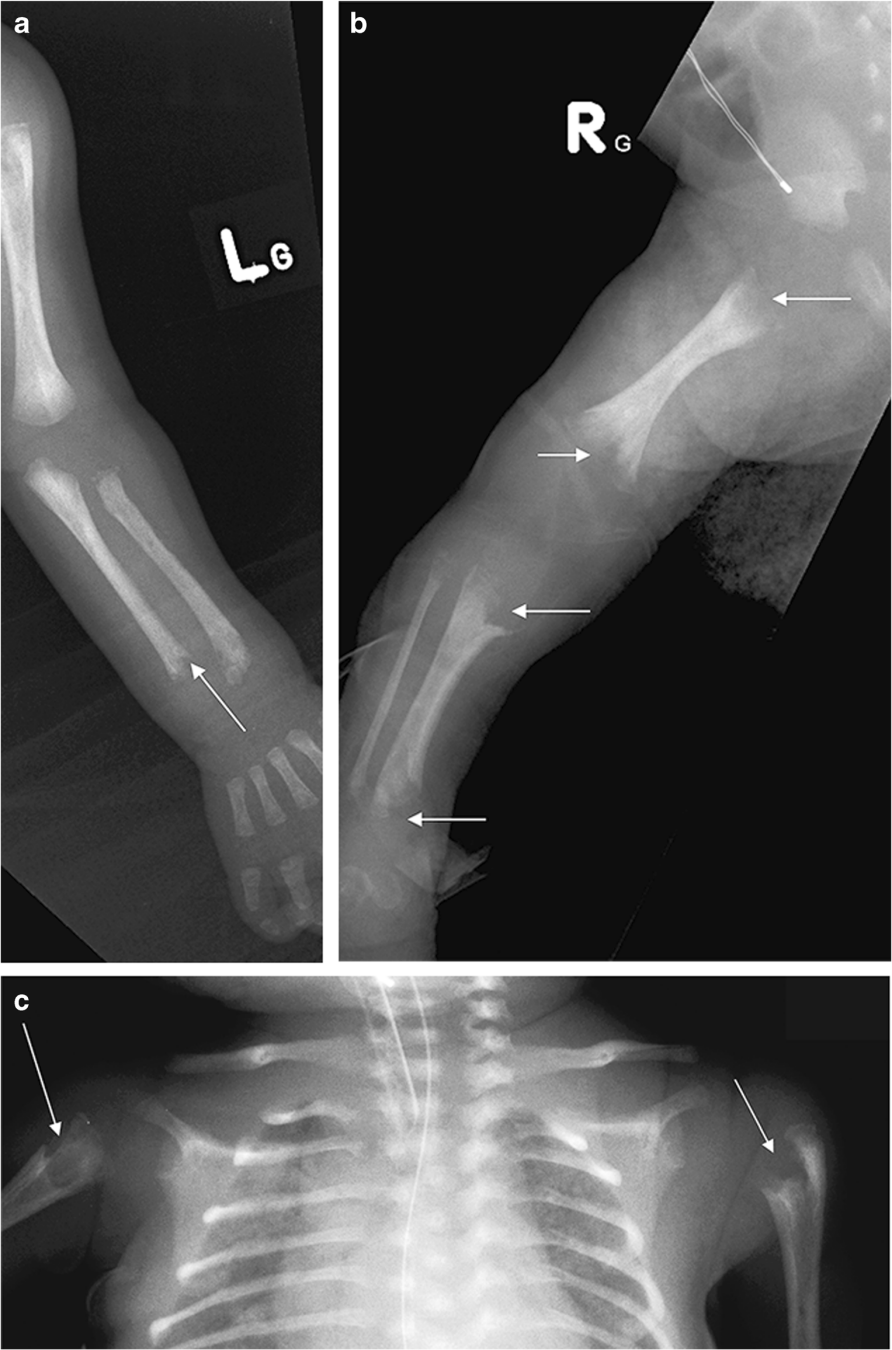
Imaging features of hypophosphatasia in a 3-week-old girl who died with HPP who died within the first 3 months of life*.***a**-**c** Anteroposterior radiographs show metaphyseal “tongues” of radiolucency (*arrow*) in the left upper limb (**a**), wide irregular metaphyses (*arrows*) and absent ossification of epiphyses of the knee in the right lower limb (**b**), and slender ribs and metaphyseal “tongues” of radiolucency (*arrows*) in the upper chest (**c**)

**Fig. 8:**
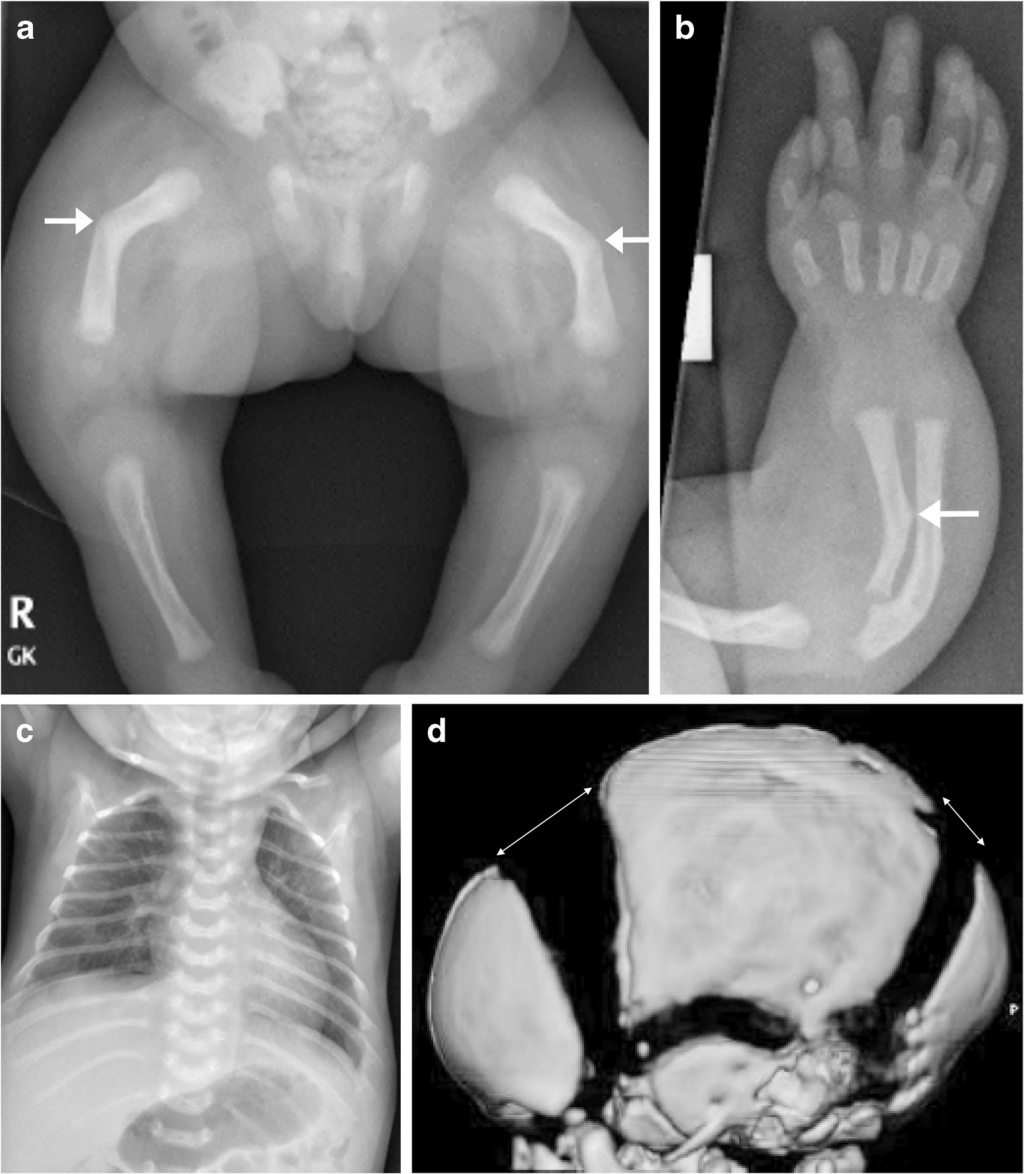
Imaging features of hypophosphatasia in a 3-month-old girl. **a**-**c** Anteroposterior radiographs show short bowed femora (*arrows*) (**a**), short bowed radius and ulna with spurred radius (*arrow*) of the right forearm (**b**), and slender, poorly ossified ribs in the chest (**c**). **d** Three-dimensional CT reconstruction in the same child shows wide sutures and fontanelles (*double-headed arrows*). [Images reproduced with permission from Radcliffe Publishing [38], pages 375–376, case 12, images 12n, 12p, 12q, and 12s]

A broad spectrum of skeletal characteristics is consistent with perinatal HPP in fetuses and neonates but is not exclusive to this disease (Table 1) (Figs. 4, 5, 6, 7 and 8) [47–58]. Early scans may appear unremarkable simply because of the normal absence of bony ossification at earlier gestational ages (<8 weeks) [28], while later scans may show characteristic features of HPP (Figs. 4, 5, 6, 7 and 8). To our knowledge, absent ossification of whole bones at or after 11 weeks’ gestation is characteristic of severe lethal HPP and is associated with very few other conditions. Shortening, bowing and angulation of the long bones are common characteristics but do not occur in all cases of HPP and are not unique to HPP. Slender, poorly ossified ribs are consistent with but not exclusive to HPP and could be related to gestational age or other conditions, in particular osteogenesis imperfecta [59, 60]. Metaphyseal lucencies or “tongues” are also strongly characteristic of but not exclusive to HPP. In HPP, the skull vault has deficient ossification with wide sutures and fontanelles; deficient ossification of the skull allows visualization of intracranial structures that are not normally visible on prenatal US [61]. Mid-diaphyseal spurs (Bowdler spurs) are rare but almost always diagnostic for HPP [10, 56, 58, 62, 63]. These spurs may protrude through or cause dimpling or indentation of the overlying skin [10, 58, 62]. Although once considered specific to HPP [62], diaphyseal spurs have also been reported in campomelic and cleidocranial dysplasia [64, 65]. Spurs can be difficult to detect on US because they are usually unossified [56], but they have been visualized as early as 18 weeks’ gestation on 3-D US when not visible on 2-D US [56, 63].

**Table 1:** Key radiographic and sonographic features of perinatal hypophosphatasia [47–58]

Long bones: shortening, bowing, angulation Small/narrow thorax (chest size smaller than abdominal circumference) Osteochondral spurs (Bowdler spur)	
Fractures	
Metaphyseal irregularities	
Lucencies (“cupping” or “tongues”)	
Ribs	
Short and beaded^a^ Thin^b^	
Deficient/absent ossification of bones	
Tubular bones, skull vault, ribs, vertebrae Abnormal sonolucency of bony structures Hypoechogenic skull Increased nuchal translucency	
Wide sutures and fontanelles^b^ Polyhydramnios	

^a^Second trimester (13–27 weeks’ gestation)

^b^Full-term neonate

The gestational age at which skeletal abnormalities are apparent in perinatal HPP varies widely, with some cases detected on prenatal US as early as 13 weeks’ gestation [66]. However, HPP diagnosis based on US findings cannot be definitive until halfway through the second trimester, when characteristics of in utero HPP become more evident. In the second trimester, all bony features become more apparent as the fetus grows and increase in visibility as ossification progresses. If the diaphysis of a tubular bone is not ossified in the second trimester, it is likely abnormal rather than physiologically related to gestational age.

Prenatal US findings may help predict lethality of a skeletal dysplasia [67]. The three most accurate predictors of fatality when evaluated in conjunction with fetal amniotic fluid volume are 3-D fetal lung volume [67, 68], the ratio of femur length to abdominal circumference and the ratio of chest circumference to abdominal circumference [67]. In general, the risk of fatality is greater if chest size is small and/or multiple rib fractures are present because this will lead to breathing difficulties ex utero [14, 67, 68]. Lethal perinatal HPP is characterized by diffuse hypomineralization of the fetal skeleton with the absence of many bones and a lack of posterior acoustic shadowing from bones that are sonographically visible [54, 67]. In particular, the neural arches and the thoracic spine may be poorly ossified or absent [56, 67]. In general, lethal perinatal HPP is clearly more severe than other forms of HPP at first detection, with a lack of improvement in skeletal signs with increasing gestational age. The severity of hypomineralization may also predict fatality. A diagnosis of lethal HPP can usually be made by the late second or early third trimester. Lack of mineralization of bones in the hands is considered an important feature. However, no correlation is apparent between the gestational age when skeletal disease is first observed and the severity of HPP after birth [66].

**Fig. 9:**
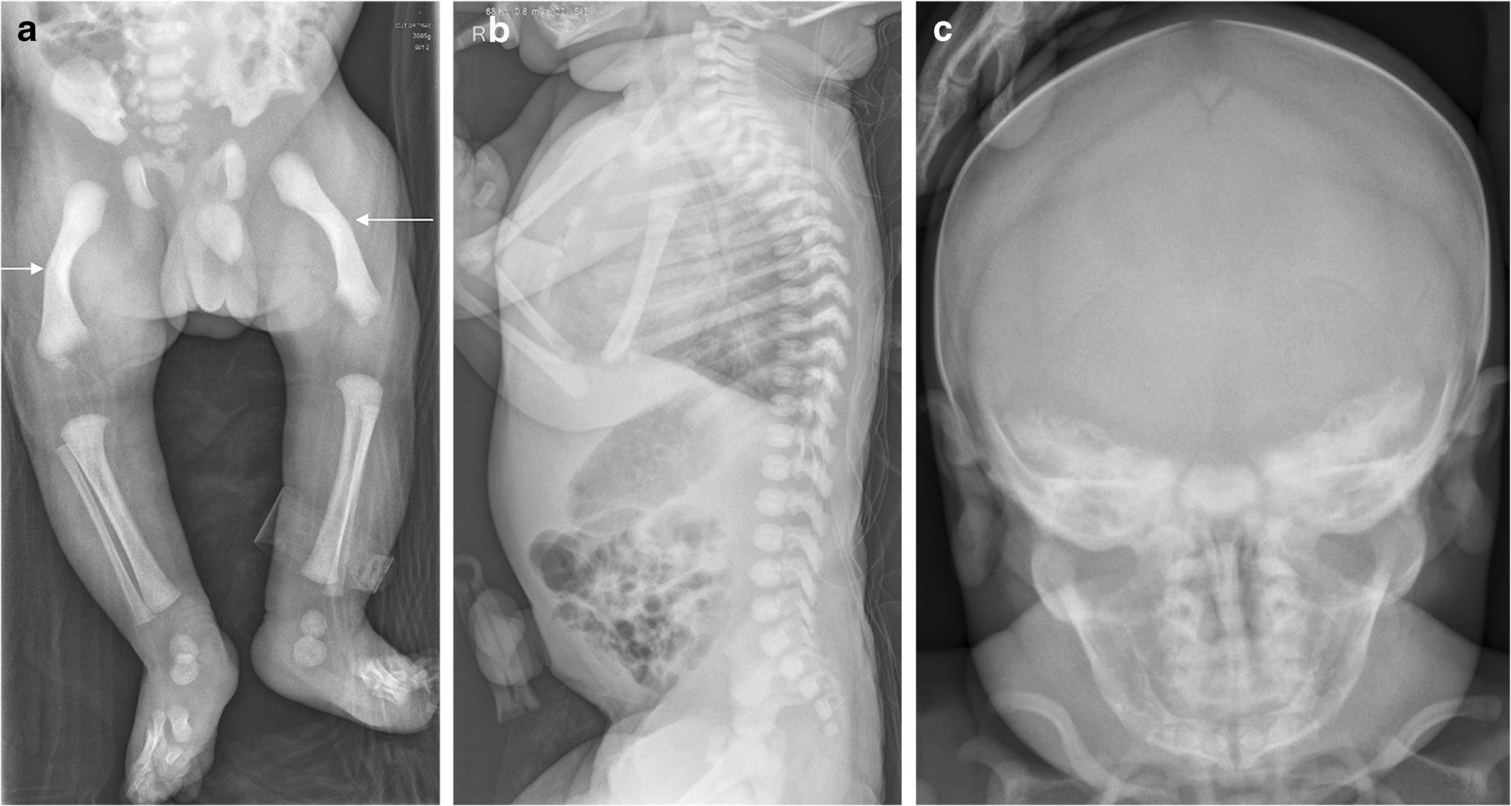
Images of a 1-day-old boy with a slowly progressing phenotype of perinatal hypophosphatasia. **a** Anteroposterior radiograph shows mild femoral bowing (*arrows*) of both lower limbs. **b,c** By comparison, the lateral spine (**b**) and anteroposterior skull (**c**) are relatively normal. The patient had low alkaline phosphatase activity and an elevated vitamin B6 concentration. Mutation of the *ALPL* gene was identified in the infant and mother. The child later had premature loss of primary dentition and femoral remodeling with growth

**Fig. 10:**
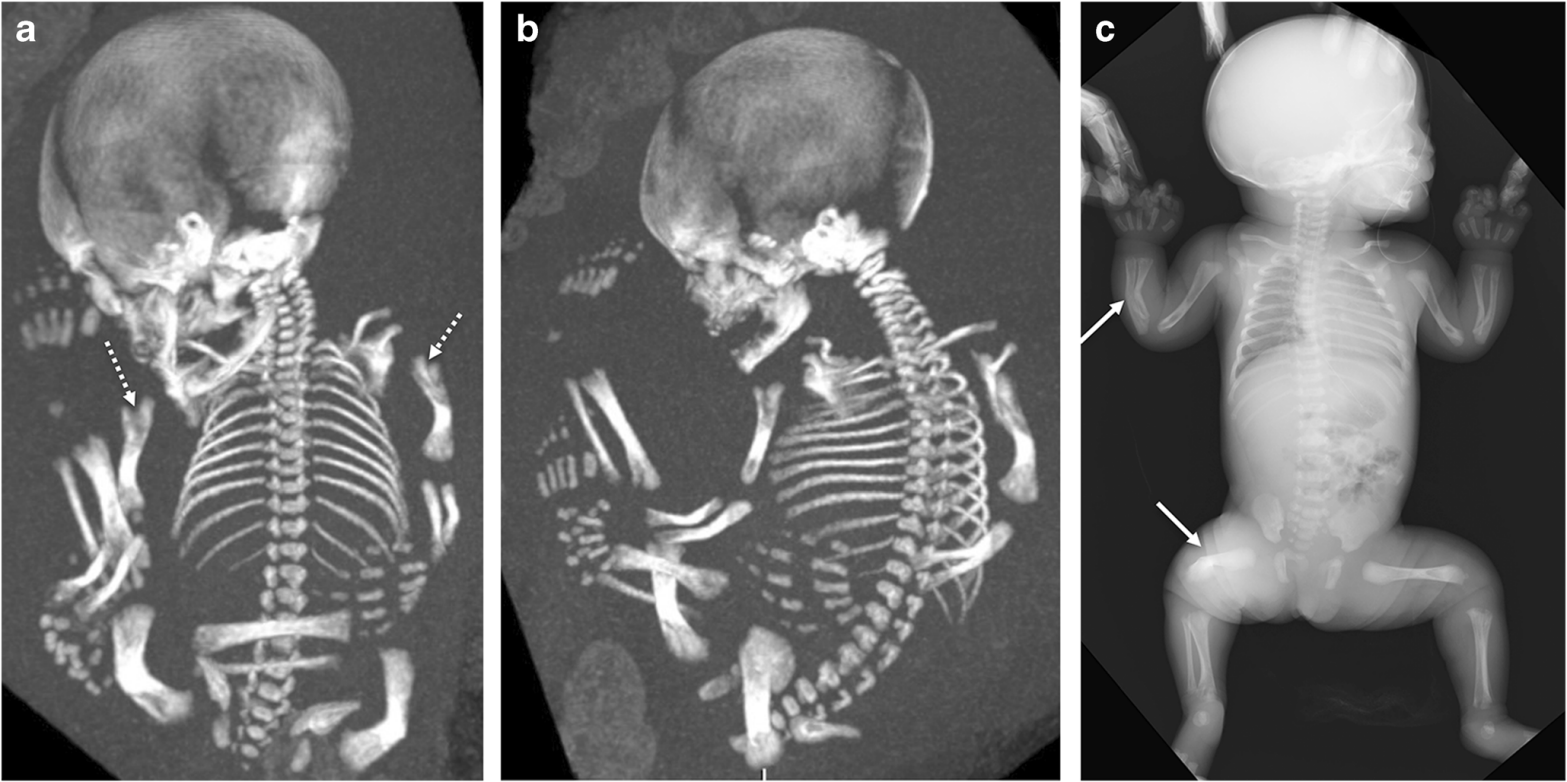
Images of a girl with a regressing phenotype of perinatal hypophosphatasia at 30 weeks’ gestation*.***a,b** Three-dimensional fetal CT scans in the coronal view (**a**) and sagittal view (**b**) show metaphyseal “tongues” of radiolucency (*dashed arrows*) and bowing of the long bones. **c** An anteroposterior radiograph obtained at birth shows regression of the metaphyseal change, although bowing persists (*arrows*)

**Fig. 11:**
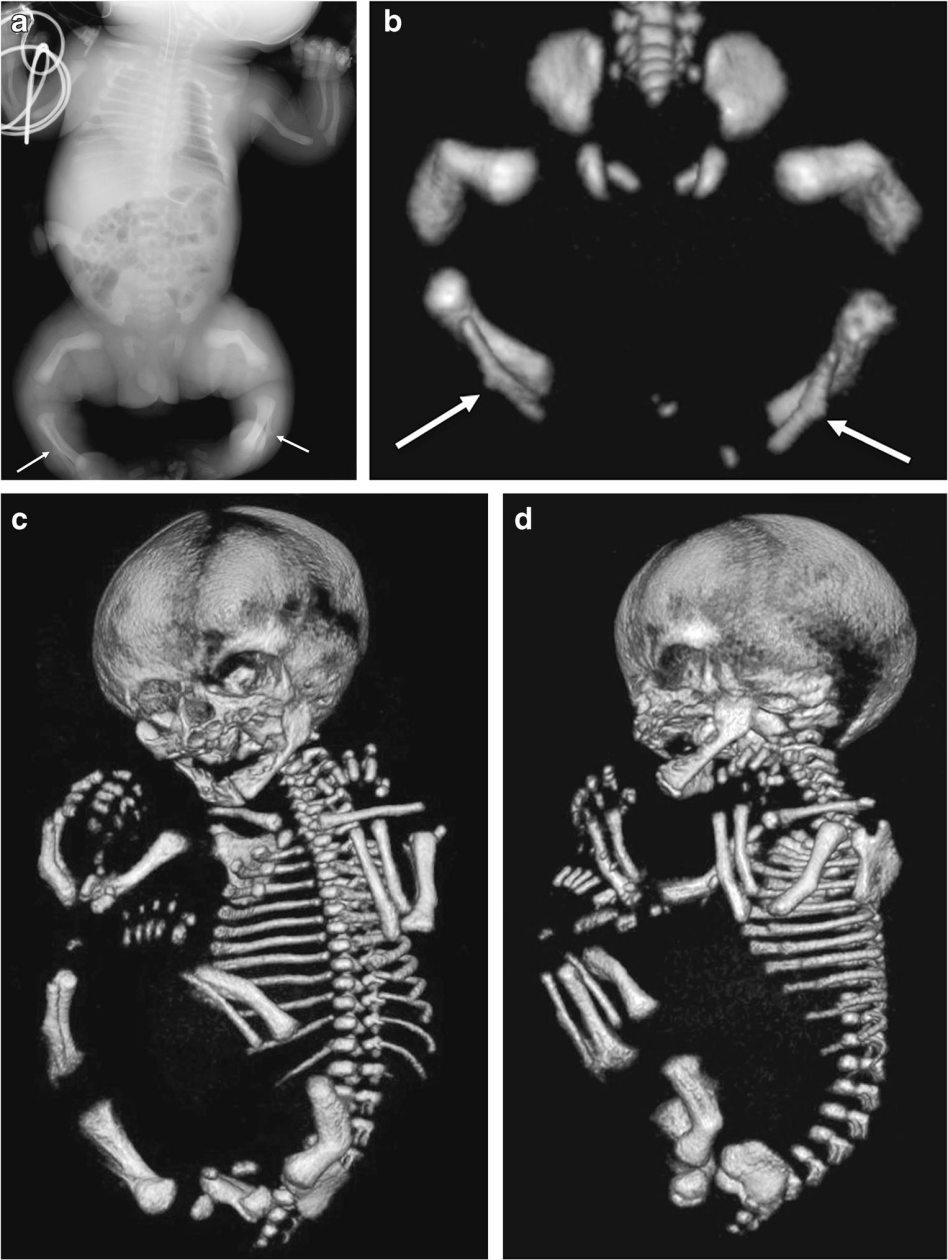
Images of a male of unknown age with benign perinatal hypophosphatasia show a less aggressive phenotype*.***a,b** Anteroposterior radiograph (**a**) and three-dimensional reconstructed CT of the lower limbs (**b**) show bowing of the long bones but normal metaphyses; note the Bowdler spurs of the fibulae (*arrows*). **c,d** Three-dimensional whole-body CT images show skull vault ossification that is within normal limits

A slowly progressing type of perinatal HPP, with only some or none of the skeletal abnormalities considered characteristic of HPP, may also present prenatally [48–50, 52, 66]. This phenotype of HPP is relatively mild at birth, with some patients presenting with long bone bowing, femoral or humeral angulation, and presumed in utero fractures but no other radiologic features of HPP (Figs. 9, 10 and 11) [49, 52, 66]. Bone ossification is usually normal or only slightly reduced on US examinations, and chest size is usually normal. In such cases, the diagnosis of HPP may be suspected based on family history (e.g., dental abnormalities) or diagnosed after confirmation of low alkaline phosphatase activity. These patients have a better prognosis in the perinatal period than patients with perinatal or infantile HPP, which may be fatal [49, 50, 52].

Pregnancy in cases of perinatal HPP may be complicated by polyhydramnios [2]. Whether perinatal HPP is associated with other maternal complications, small size for gestational age or premature birth has not been systematically studied. A retrospective review of 15 Manitoban Mennonite patients with perinatal HPP reported during an 80-year period (1927–2007) found that most of the infants (73.3% [11/15]) were born at full term, 13.3% (2/15) were born early at 36 weeks’ gestation, and 13.3% (2/15) were born prematurely at 30 and 33 weeks’ gestation [15]. Birth weights (*n*=6) ranged from below the 5th percentile (2.3 kg) to within the 25th–50th percentile (3.3 kg). As technology advances, we may learn more about maternal complications.

### Differential diagnosis

Metaphyseal abnormalities similar to those observed in HPP are also observed in rickets and osteopathy of prematurity [69]. Active rickets may present with widened zones of provisional calcification and wide costochondral junctions, including widening along the anterior ends of the ribs (i.e. rachitic rosary). Osteopathy of prematurity is associated with radiologic changes characteristic of rickets, and fractures may be seen in infants with very low birth weights [69, 70].

## Osteogenesis imperfecta

**Fig. 12:**
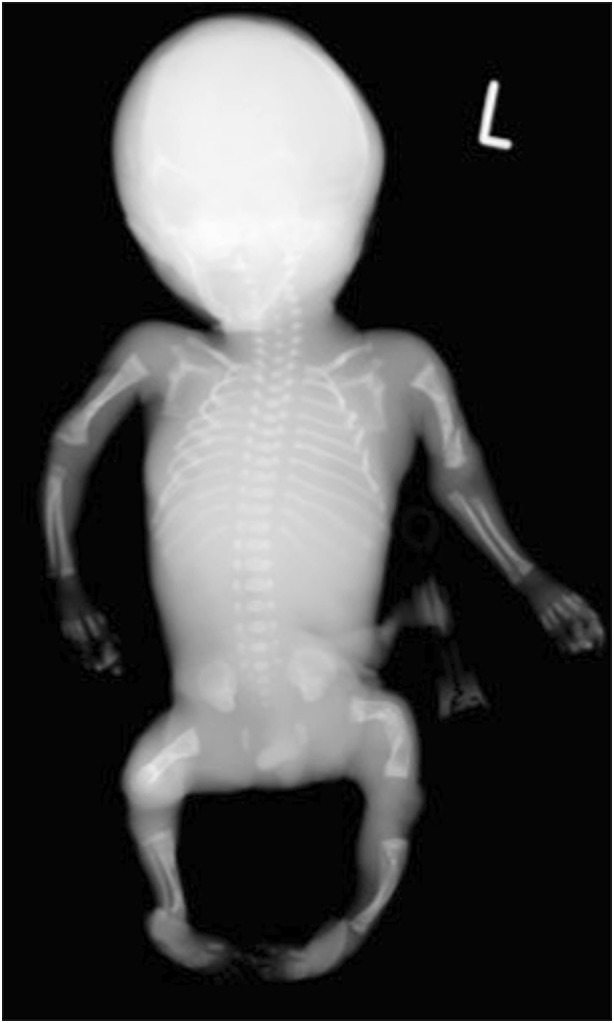
Radiographic features in a 22 weeks’ gestation male fetus with lethal osteogenesis imperfecta type II*.* Anteroposterior babygram shows generalized osteopenia, deformities of the ribs, and absent ossification of the skull vault

**Fig. 13:**
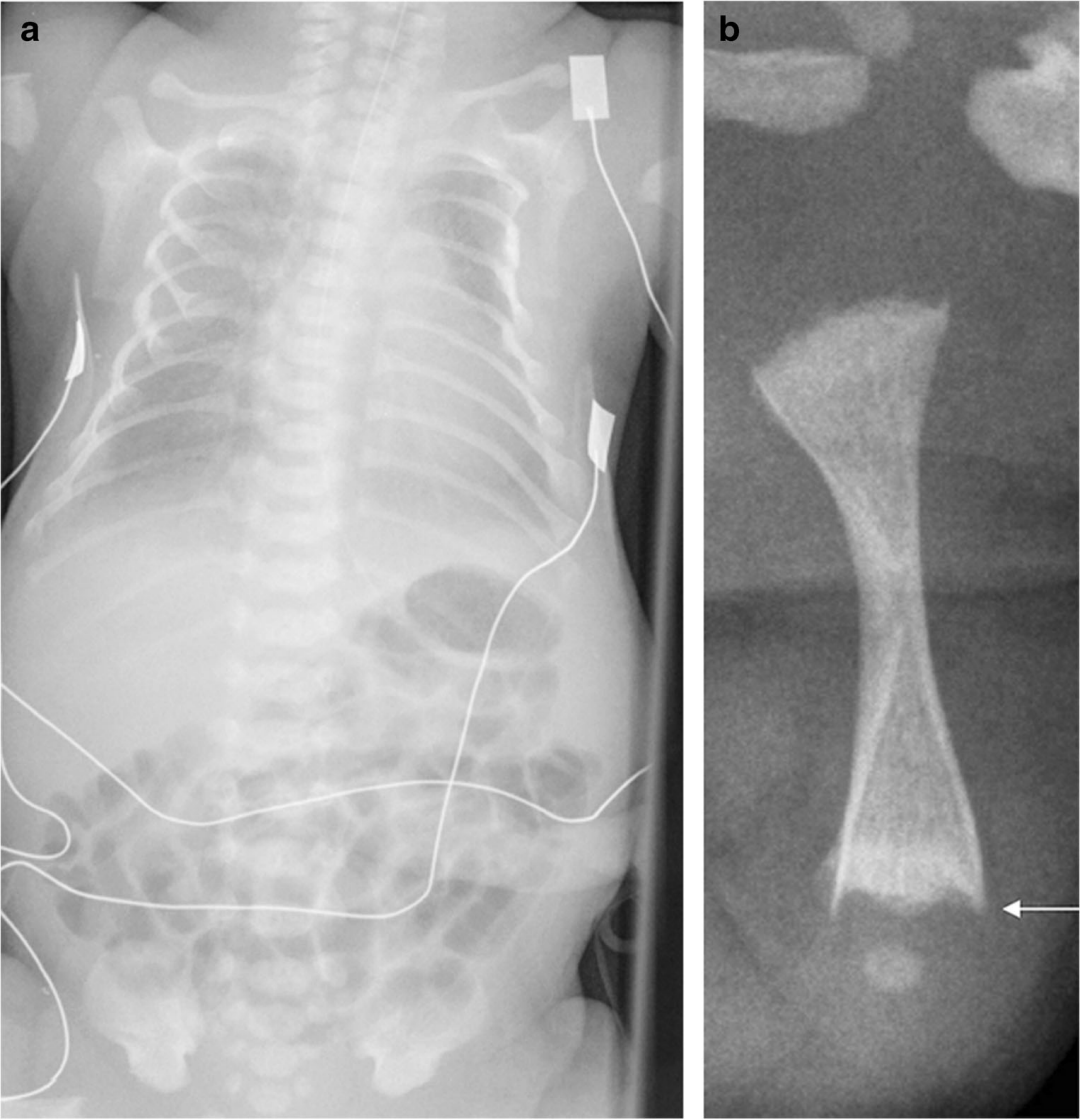
Radiographic features of a girl with osteogenesis imperfecta type V*.***a,b** Anteroposterior radiographs show slender, deformed ribs of the chest and abdomen on day 1 (**a**) and spurred, sclerotic and flared metaphyses (*arrow*) of the left femur at 6 weeks of age (**b**). [Images reproduced with permission from Radcliffe Publishing [38], page 365, case 1, images 1c and 1e]

**Fig. 14:**
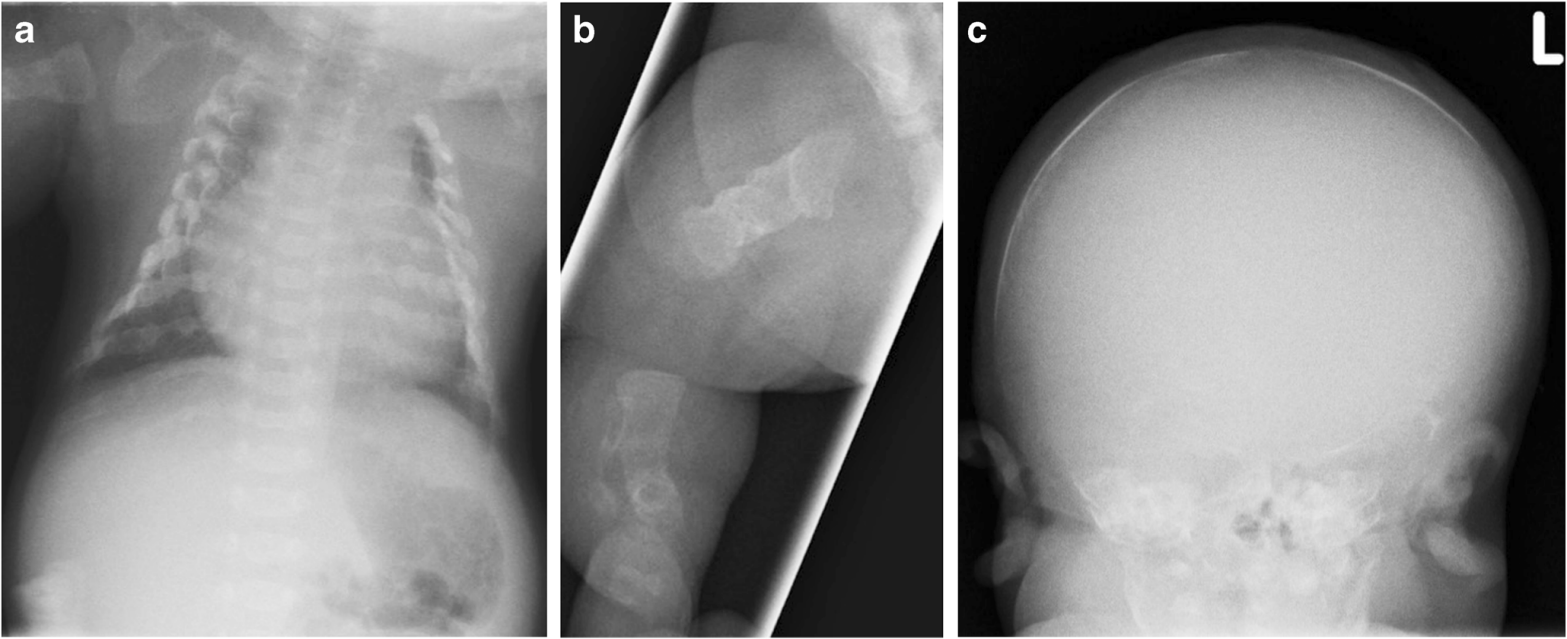
Radiographic features of an infant of unknown gender at birth (delivered at term) with severe osteogenesis imperfecta type III. **a**-**c** Anteroposterior radiographs show broad ribs with multiple fractures in the chest (**a**), broad tubular bones with multiple fractures of the right lower limb (**b**) and absent ossification of the skull vault (**c**)

**Fig. 15:**
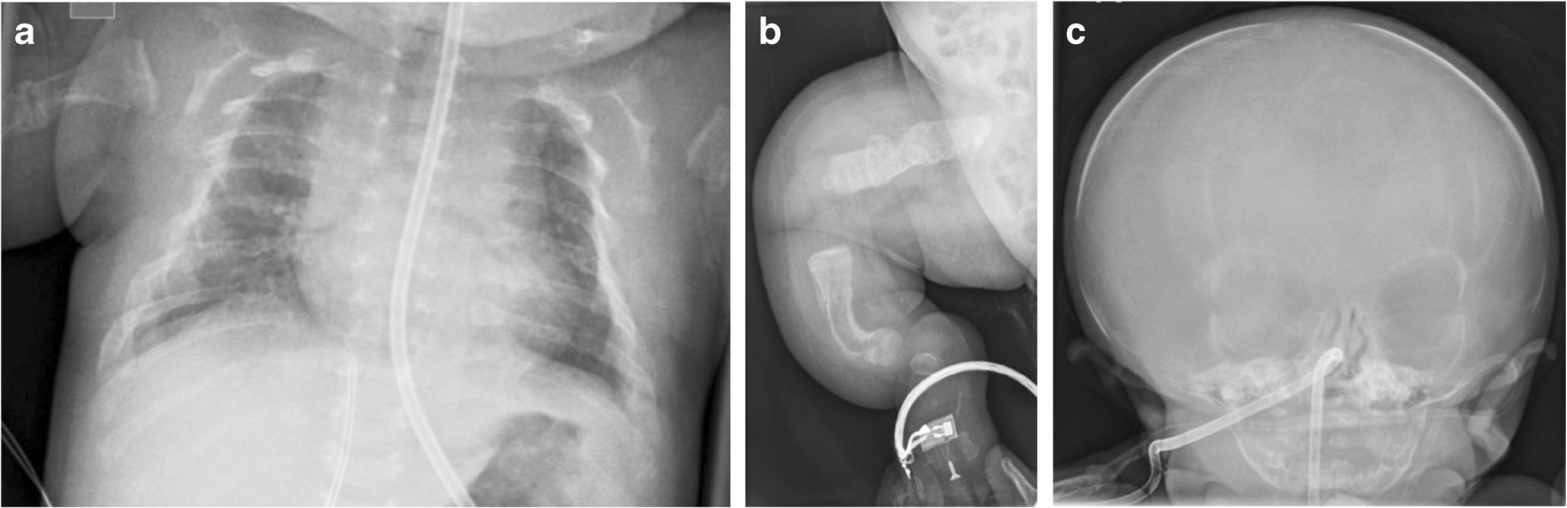
Radiographic features of a 2-week-old infant of unknown gender with severe osteogenesis imperfecta type III. Anteroposterior radiographs show broad ribs with multiple fractures (‘‘beading”) in the chest (**a**), broad tubular bones with multiple fractures and bowing of the tibia and fibula of the right lower limb (**b**), and deficient ossification of the skull vault (**c**; less severe than in the infant shown in Fig. 14)

Osteogenesis imperfecta and perinatal and infantile HPP share features of reduced bone density, deficient ossification of the skull vault, bowed long bones, fractures, gracile ribs and narrow thorax (Table 2) [27, 29, 56, 59–61, 64, 65, 71–77]. Although it may be difficult to distinguish osteogenesis imperfecta from HPP on US [27], certain patterns of demineralization may help [61]. Osteogenesis imperfecta types II, III and IV are characterized by overall diffuse osteopenia (Figs. 12, 13, 14 and 15), whereas HPP is characterized by a near complete lack of mineralization in individual bones with more densely or normally mineralized adjacent bones [61, 73, 74, 78]. Wormian bones of the skull and compression fractures in the spine are common findings in the majority of cases of severe osteogenesis imperfecta [59, 79] but not in HPP. Demineralization of the skull is usually severe and diffuse in HPP. This is in contrast to the “island-like” centers of ossification (i.e. Wormian bones) in the frontal, parietal and occipital bones often observed in osteogenesis imperfecta. The hand bones are echogenic in osteogenesis imperfecta but are usually sonolucent in HPP [54, 61]. Similar to HPP, in the neonatal period, osteogenesis imperfecta type V may present with reduced bone density, metaphyseal widening/flaring and widening of the growth plates [59]. However, unlike active rickets, the metaphyses are sclerotic and irregular and there may be centrally located wedge-shaped sclerosis of the anterior vertebral bodies and unusual lucency of the metadiaphyseal regions [59].

**Table 2:** Differential diagnoses for perinatal hypophosphatasia (HPP)

	Perinatal hypohposphatasia [29, 61, 75]	Osteogenesis imperfecta type II [56, 59, 61, 71]	Campomelic dysplasia [27, 29, 65, 76]	Achondrogenesis/hypochondrogenesis [27, 29, 71]	Cleidocranial dysplasia [29, 64, 77]	Thanatophoric dysplasia [27, 29, 71]
Long bones	Micromelia Bowed Angulation (tibiae) Absent ossification of whole bones Diaphyseal spurs (tibial dimple) Metaphyseal lucencies (“tongues”) Hypoplastic fibulae	Micromelia Thick Crumpled shafts Multiple fractures Callus formation Wrinkled appearance on sonograms	Shortening of the long bones Angulation (tibiae and short fibula) Mild to moderate bowing Diaphyseal spurs (tibial dimple) Hypoplastic fibulae	Micromelia Metaphyseal spikes Dysplastic limbs	Bowed limbs Diaphyseal spurs	Micromelia Femoral bowing (type I)
Thorax	Shortened ribs Thin ribs Small thoracic circumference	Shortened ribs Thick/broad irregular ribs Continuous beading of ribs Flaring at anterior rib ends Small chest circumference	11 pairs of ribs Absent or hypoplastic scapula Small, bell-shaped thorax Short ribs	Narrow barrel-shaped thorax Prominent abdomen	Hypoplastic or absent clavicles	Shortened ribs Narrow thorax Prominent abdomen
Skull	Absent ossification of skull base Wide sutures and fontanelles Prominent falx cerebri in sonograms Caput membranaceum	Wormian bones Hypomineralization of skull Abnormal skull shape Caput membranaceum Flattened facial profile		Severe hypomineralization of skull Micrognathia Flattened facial profile	Hypomineralization of the skull Wide sutures and fontanelles Parietal bones absent in extreme cases	Macrocephaly Craniosynostosis (type II) Narrowed or closed cranial sutures Metopic bossing
Spine	Underossification of vertebrae and neural arches Absent pedicles and bodies of the vertebrae	Increased nuchal translucency	Absent pedicles of the T spine Scoliosis	Severe hypomineralization of vertebral bodies Platyspondyly Large nuchal lucencies		Platyspondyly
Pelvis	Absent ossification		Dislocated hips	Crescent ilia Absent ischia	Deficient ossification of the pubic rami Wide symphysis pubis	Trident acetabulum
Hands and feet	Underossification and sonolucency of hands	Hands appear grossly normal and are echogenic	Short fingers and toes Clubbed feet	Very small hands and feet Trident hands		Brachydactyly
Other	Generalized hypomineralization Decreased echogenicity of bones Small for gestational age Polyhydramnios	Generalized hypomineralization	Normal bone density Micrognathia Flat nasal bridge Low-set ears Nuchal edema	Early hydrops Cystic hygroma Short crown–rump length Polyhydramnios (type II)	Omphalocele	Normal trunk length Depressed nasal bridge Hydrocephaly Polyhydramnios

## Campomelic dysplasia

**Fig. 16:**
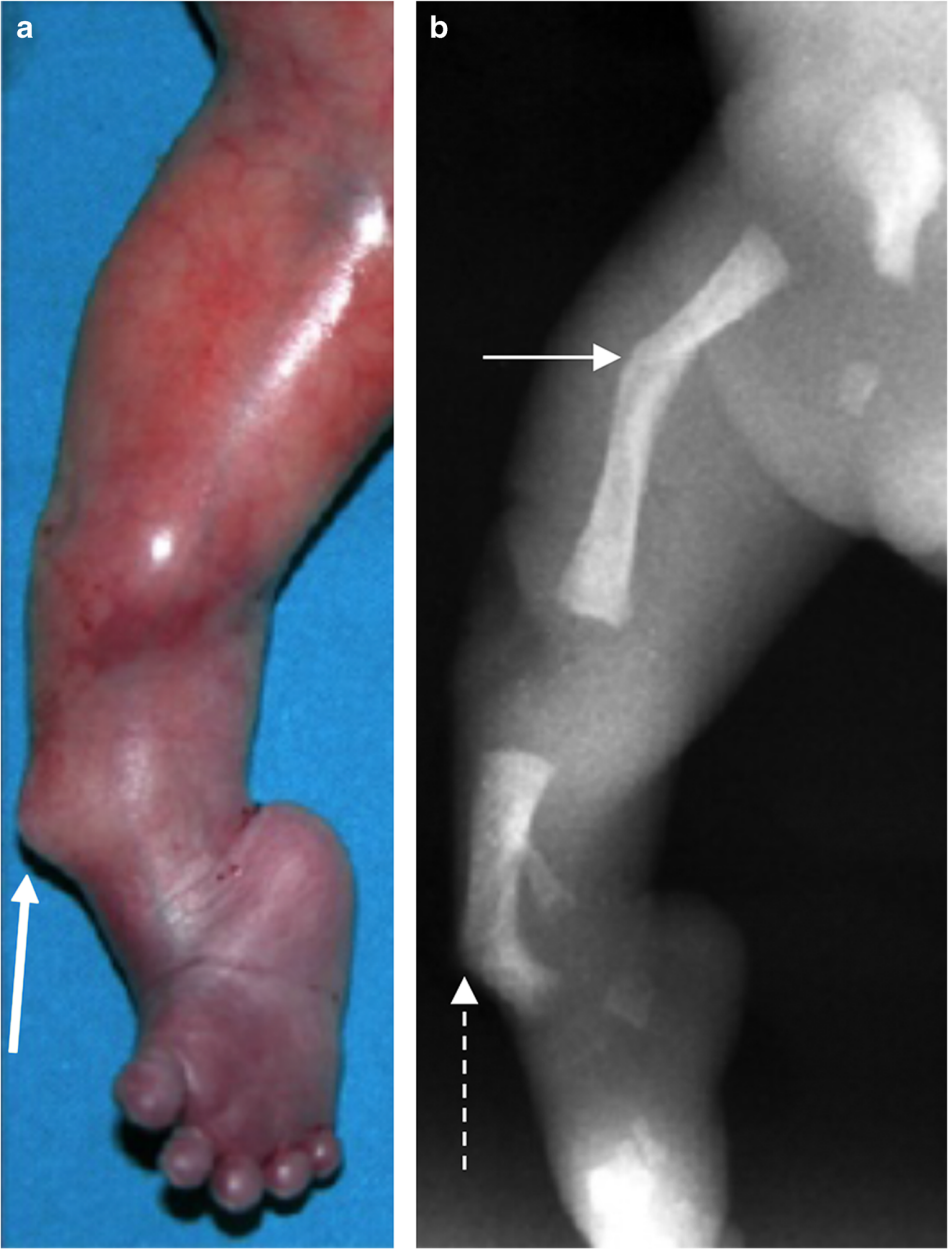
Clinical and radiographic features of lethal campomelic dysplasia in a male neonate. **a,b** Photograph (**a**) and radiograph (**b**) show angulation at the junction of the proximal third and distal two-thirds of the femur (*arrow*) and the junction of the proximal two-thirds and the distal third of the tibia (*dashed arrow*). Note the clinical spur (*yellow arrow*), which may also be seen in hypophosphatasia. [Images reproduced with permission from Radcliffe Publishing (38), page 246, case 1, image 1c]

**Fig. 17:**
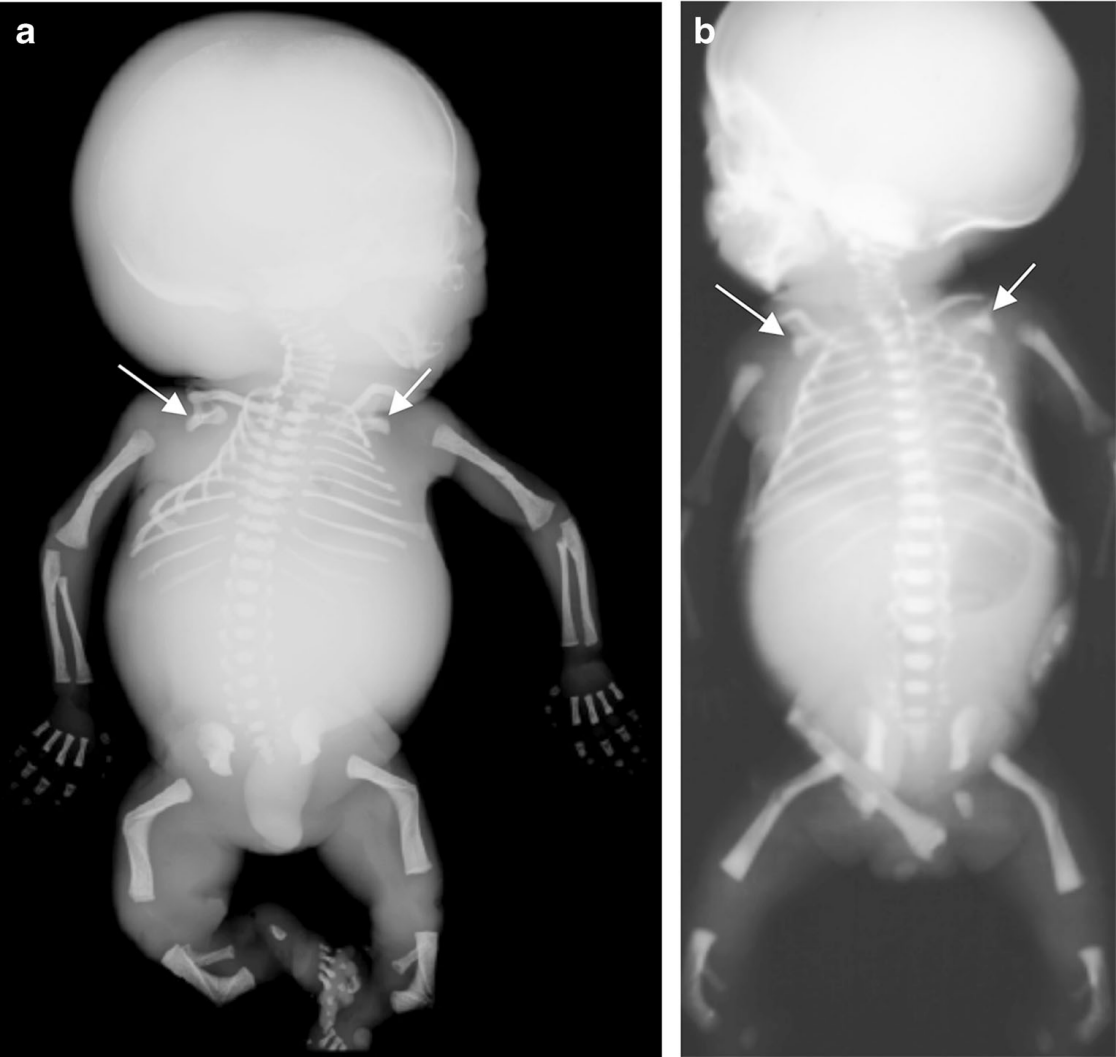
Radiographic features of lethal campomelic dysplasia in female and male fetuses*.***a,b** Anteroposterior radiographs in female (**a**) and male (**b**) fetuses show 11 pairs of ribs, hypoplastic scapulae (*arrows*), absent ossification of thoracic pedicles, characteristic bowing/angulation of tibiae and fibulae, and narrow iliac wings. [Image (**a**) reproduced with permission from Radcliffe Publishing [38], page 249, case 13, image 13c]

Campomelic dysplasia shares some characteristics with HPP, including shortening, bowing and angulation of the long bones, diaphyseal spurs, tibial dimple, absent ossification of the pedicles and hypoplastic fibulae (Table 2) (Figs. 16 and 17) [27, 65, 76]. Unlike HPP, the absence of ossification of the pedicles is limited to the thoracic spine in campomelic dysplasia. Campomelic dysplasia is also distinguished from HPP by characteristic sites of long bone angulation, specifically in the femur at the junction of the proximal third and distal two-thirds and in the tibia at the junction of proximal two-thirds and distal third. Other distinguishing characteristics of campomelic dysplasia include the absence of ossification of the wings of the scapulae, dislocated elbows, 11 pairs of ribs, narrow iliac wings and normal bone density [27].

## Achondrogenesis/hypochondrogenesis

**Fig. 18:**
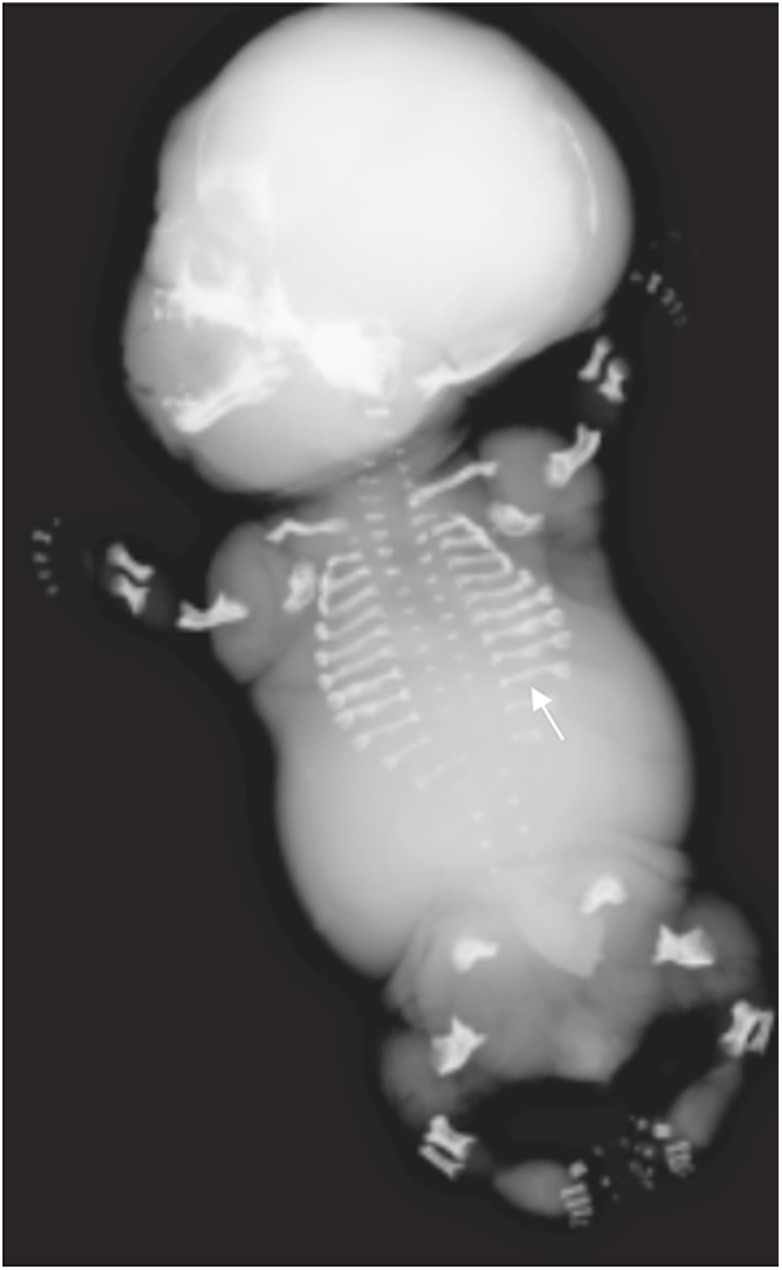
Radiographic features in a female fetus with achondrogenesis type IA*.* Anteroposterior radiograph in the most severe form of the disease shows short limbs, short ribs, deficient ossification of the pelvis and skull, and “beaded” ribs due to healing fractures (*arrow*). [Image reproduced with permission from Radcliffe Publishing [38], page 215, case 3, image 3b]

**Fig. 19:**
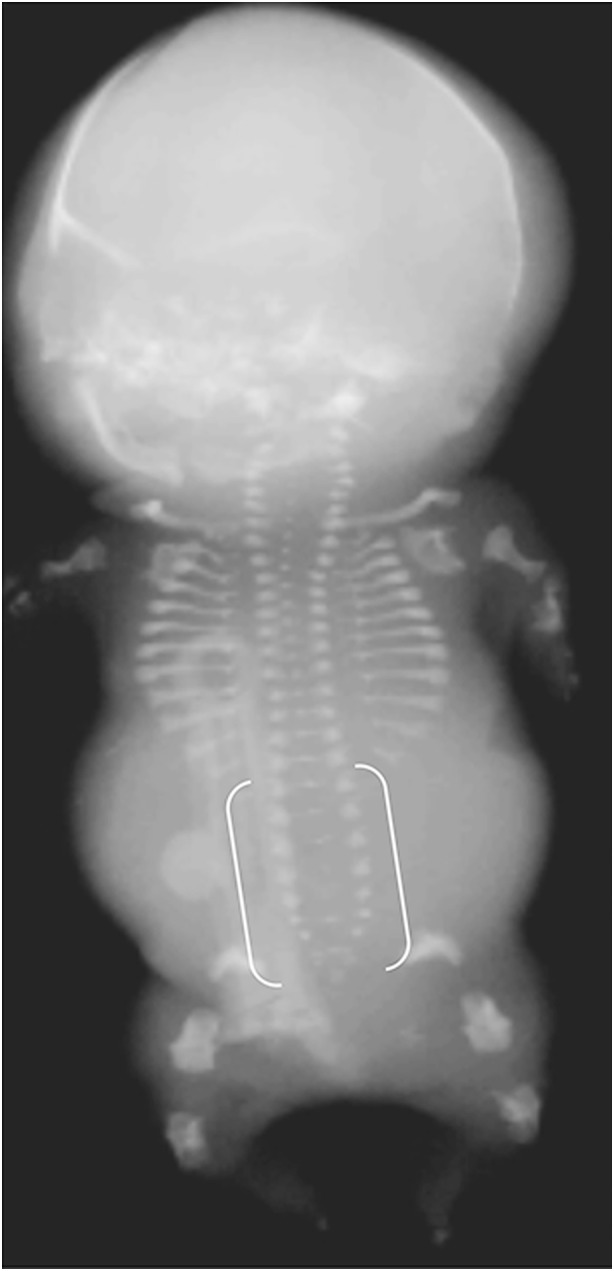
Radiographic features in a female fetus with achondrogenesis type IB*.* Anteroposterior radiograph shows short ribs, short limbs, deficient ossification of the ischia and widening of the lumbar interpedicular distances (*brackets*), which has been likened to the shape of a cobra’s head. [Image reproduced with permission from Radcliffe Publishing [38], page 106, case 3]

**Fig. 20:**
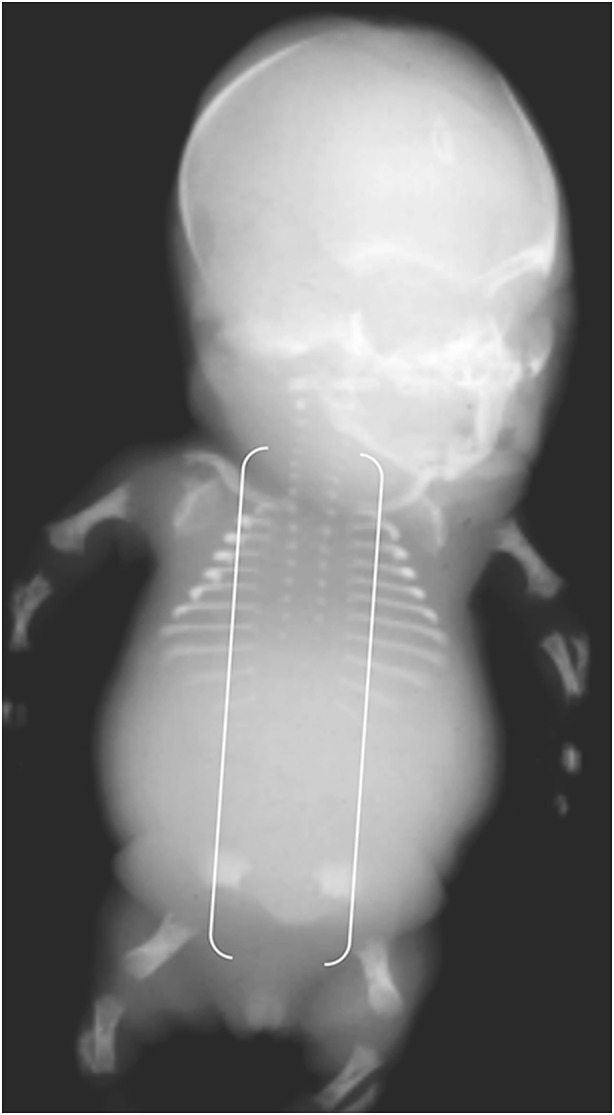
Radiographic features of a male fetus with achondrogenesis type II*.* Anteroposterior radiograph of the least severe form of the disease shows less marked shortening of ribs and long bones, and improved length of long bones and pelvic ossification; however, there is absent ossification of all vertebral bodies and of the lower thoracic, lumbar and sacral pedicles (*brackets*). [Image reproduced with permission from Radcliffe Publishing [38], page 65, case 4]

Achondrogenesis is characterized by early hydrops and a short trunk (crown–rump length), narrow barrel-shaped thorax and prominent abdomen (Figs. 18, 19 and 20) [27]. Achondrogenesis types IA/B are inherited by autosomal-recessive transmission and are associated with extreme micromelia, short hands and feet, poor mineralization, a large head, a flat face and a short neck. Achondrogenesis type II (autosomal dominant) is less severe and presents later in gestation than type I, often with polyhydramnios. Hypochondrogenesis is characterized by a small thorax, short limbs, a flat face with micrognathia, a short trunk and macrocephaly, a flat nose and depressed nasal bridge [27].

In achondrogenesis and hypochondrogenesis, deficient ossification of the vertebral bodies is usually most severe in the lumbosacral and cervical spine [56]. In achondrogenesis, the whole spine may be unossified, with complete absence of vertebral bodies. In contrast, HPP typically presents as deficient spine ossification in the thoracic region, with a sharp demarcation between almost normal ossification in the lumbar spine and complete absence of ossification in the thoracic spine.

## Cleidocranial dysplasia

**Fig. 21:**
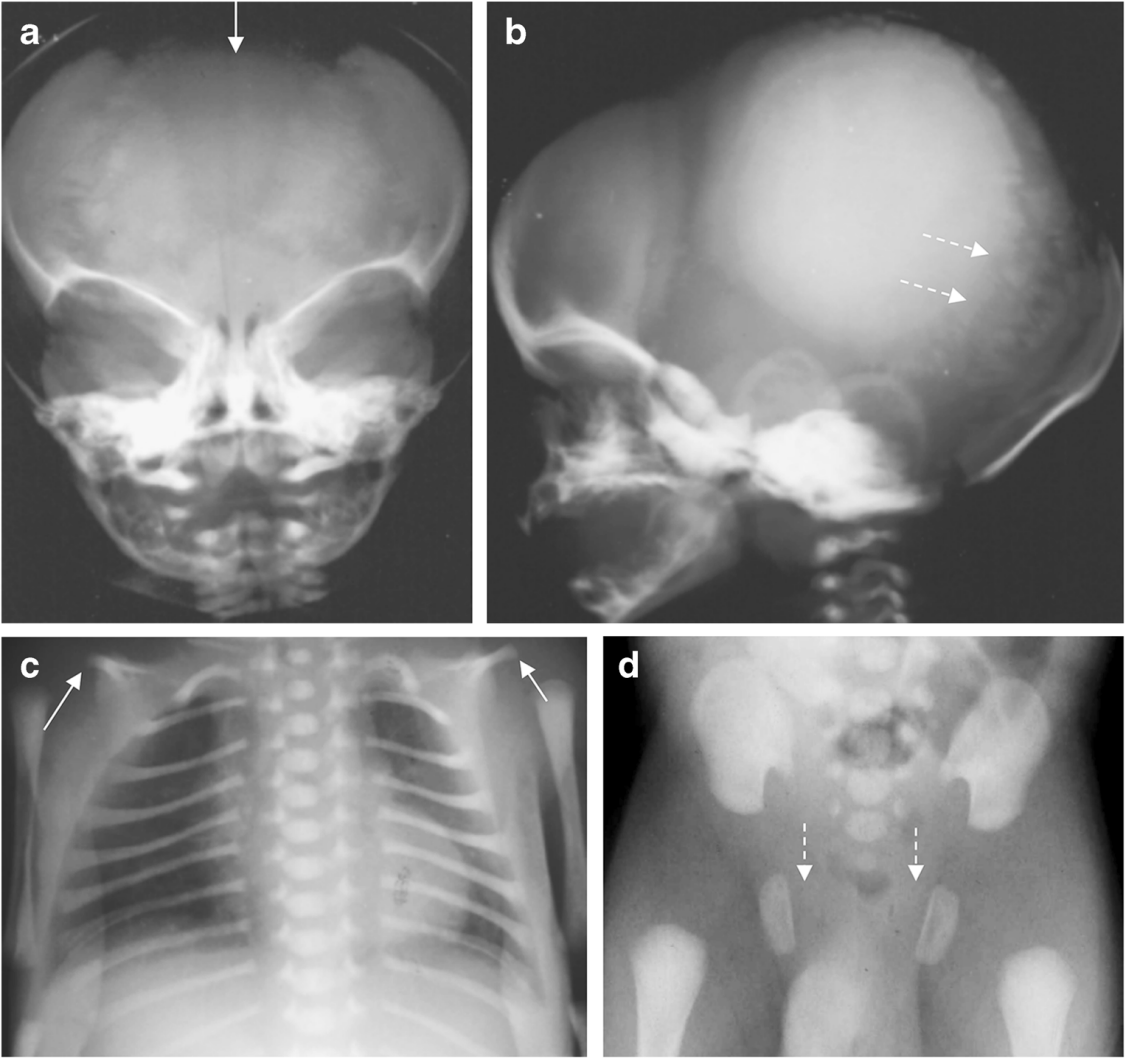
Radiographic features of cleidocranial dysplasia. **a,b** Radiographs of the skull in the anteroposterior view (**a**) and lateral view (**b**) show wide sutures and fontanelles (*arrow*) and multiple Wormian bones (*arrows*). **c** Anteroposterior chest radiograph shows hypoplastic clavicles (*arrows*). **d** Anteroposterior pelvic radiograph shows deficient ossification of the pubic bones (*arrows*). [Images reproduced with permission from Radcliffe Publishing (38), page 390, case 4, images 4a-b, and case 3, images 3b-c]

Cleidocranial dysplasia is an autosomal-dominant skeletal dysplasia characterized by clavicular hypoplasia or aplasia, delayed closure of fontanelles and sutures, and hypoplasia of the pubic bones (Table 2) (Fig. 21) [64]. Prenatal US may reveal absent or hypoplastic clavicles, missing nasal bones, and hypomineralization of the cranium and vertebral spine early in the second trimester [80–82]. Later in life, patients with cleidocranial dysplasia may have dental anomalies (e.g., delayed eruption of primary and secondary dentition, supernumerary teeth) and short stature. One case of cleidocranial dysplasia misdiagnosed as HPP during infancy has been reported [64]. Some patients with severe cleidocranial dysplasia may have low serum alkaline phosphatase activity [83, 84]. However, these patients may also have normal serum pyridoxal 5′-phosphate and urine phosphoethanolamine [83, 84].

## Thanatophoric dysplasia

**Fig. 22:**
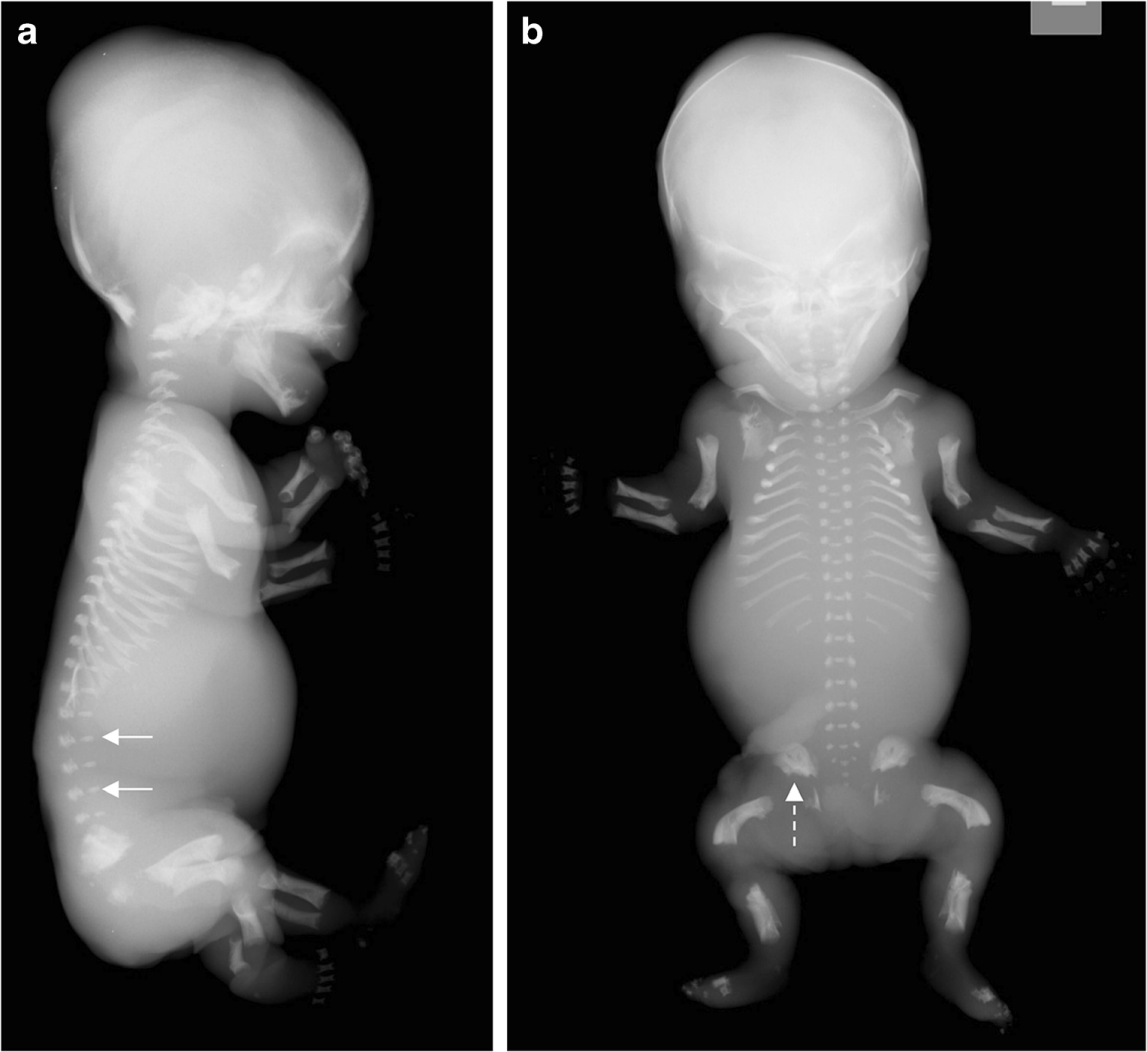
Radiographic features of a 21 weeks’ gestation male fetus with thanatophoric dysplasia type 1*.***a** Lateral radiograph shows significant platyspondyly (*arrows*), with bowing of the humeri, femora and short ribs and a small thorax. **b** An anteroposterior radiograph shows horizontal acetabula (*dashed arrow*)

Thanatophoric dysplasia is one of the most commonly encountered lethal prenatal skeletal dysplasias [27]. Characteristic in utero sonographic features of thanatophoric dysplasia include severe micromelia and brachydactyly, bowed (type I) or straight (type II) long bones, severe platyspondyly with normal trunk length, narrow thorax, short ribs and prominent abdomen apparent by the 18-week morphology US (Table 2) (Fig. 22) [27]. In one report, suspicious findings on US performed at 13 weeks’ gestation prompted a repeat scan at 15 weeks to confirm the diagnosis of thanatophoric dysplasia [85].

### Suspicion of hypophosphatasia: Next steps

If perinatal HPP is suspected, alkaline phosphatase activity may be assessed in umbilical cord blood or chorionic villus samples [86–89]. Chorionic villus sampling for assay of alkaline phosphatase activity has been used to diagnose HPP as early as 11–12 weeks’ gestation [89, 90]. In some cases, however, alkaline phosphatase activity may fail to identify affected fetuses, as alkaline phosphatase activity varies with gestational age [89, 90]. After 13 weeks’ gestation, the placenta may produce alkaline phosphatase, affecting the interpretation of the analysis.

Measurement of parental serum alkaline phosphatase activity can be useful for prenatal diagnosis of HPP [87]. A retrospective analysis of 77 cases of fetal skeletal dysplasia (including 17 of HPP) in Japan from 2007 to 2016 showed that the presence of at least one abnormally low maternal (<123 IU/L) or paternal alkaline phosphatase value (<165 IU/L) any time during pregnancy had high sensitivity (82%) specificity (75%), and positive predictive value (80%) for HPP [87].

For newborns, alkaline phosphatase activity must be compared with age- and gender-adjusted reference ranges for the testing laboratory; alkaline phosphatase reference ranges vary widely depending on patient age and gender and the laboratory and methods used [75]. If alkaline phosphatase activity is low or suspicion for HPP is high based on images, additional testing may be necessary. In newborns, these tests should include urine concentrations of phosphoethanolamine, and serum concentrations of pyridoxal 5′-phosphate (i.e. vitamin B6), calcium, vitamin D and parathyroid hormone [75].

Genetic testing for *ALPL* mutations can be confirmatory in cases of diagnostic uncertainty [91, 92]. However, clinicians should be aware of the depth of coverage with whole-exome sequencing and next-generation sequencing technology, as some pathogenic variants may not be detected [92, 93].

### Medical genetic evaluation and genetic counseling

A medical genetic evaluation should be obtained when a diagnosis of HPP is being considered. In many centers, this will be through a prenatal genetics clinic. A medical genetics physician or genetics counselor can help obtain gene testing and interpret results, especially if variants of unknown significance are found. If gene testing results are normal, alternative genetic causes for the apparent bony abnormalities can be pursued in consultation with the radiologist and obstetrician. Family genetic counseling should also begin when HPP is suspected, and a detailed pedigree should be obtained. Genetic counseling in cases of suspected HPP may be particularly difficult due to the autosomal-dominant and autosomal-recessive patterns of inheritance and the phenotypic heterogeneity of the disease [94]. The family should receive counseling by a clinician familiar with the treatment of children with HPP so that they are informed on available treatment options before making decisions about terminating the pregnancy.

Prompt diagnosis of HPP is important, as the health care provider must evaluate treatment strategies for newborns with HPP as early as possible. If the decision is made to treat, treatment should begin as early as possible postnatally. All decisions must be made in consultation with the parents. It is important to assemble a multidisciplinary team for care in the perinatal period. The team should include the following specialists: neonatologist, pediatrician, geneticist, endocrinologist, radiologist, nephrologist, specialist nurses, genetic counselor, social worker, physical therapist, occupational therapist, respiratory physician (especially if long-term ventilation is needed), and an ears, nose, and throat specialist, neurologist and craniofacial surgeon (if craniosynostosis is present). Neonatologists should be prepared to provide invasive respiratory support, as many babies born with skeletal dysplasias are likely to require ventilation.

## Conclusion

Perinatal HPP is associated with a broad spectrum of imaging findings that overlap with other perinatal skeletal dysplasias. Certain features (e.g., sites of angulation and hypomineralization, spurs, metaphyseal characteristics) may help distinguish HPP from other skeletal dysplasias. *ALPL* gene mutation testing during pregnancy can confirm the diagnosis before delivery. Alkaline phosphatase results are essential for confirming a suspected diagnosis of perinatal HPP. Early recognition of the disease provides more opportunity for education and counseling to prepare the parents, allows for the assembly and preparedness of a multidisciplinary care team upon delivery, and provides additional time to consider and discuss treatment options, with the goal of improving duration and quality of life or minimizing unnecessary suffering for the affected child and the family.

## References

[1] Weiss MJ, Cole DE, Ray K (1988). A missense mutation in the human liver/bone/kidney alkaline phosphatase gene causing a lethal form of hypophosphatasia. Proc Natl Acad Sci U S A.

[2] Whyte MP (2018) Hypophosphatasia and how alkaline phosphatase promotes mineralization. In: Thakker RV, Whyte MP, Eisman J, Igarashi T (eds) Genetics of bone biology and skeletal disease, 2nd edn. Elsevier (Academic Press, London), San Diego, CA, pp 481–504

[3] Rockman-Greenberg C (2013). Hypophosphatasia. Pediatr Endocrinol Rev.

[4] Whyte MP, Mahuren JD, Vrabel LA, Coburn SP (1985). Markedly increased circulating pyridoxal-5′-phosphate levels in hypophosphatasia. Alkaline phosphatase acts in vitamin B6 metabolism. J Clin Invest.

[5] Russell RG (1965). Excretion of inorganic pyrophosphate in hypophosphatasia. Lancet.

[6] Fleisch H, Russell RG, Straumann F (1966). Effect of pyrophosphate on hydroxyapatite and its implications in calcium homeostasis. Nature.

[7] Millan JL (2013). The role of phosphatases in the initiation of skeletal mineralization. Calcif Tissue Int.

[8] Linglart A, Biosse-Duplan M (2016). Hypophosphatasia. Curr Osteoporos Rep.

[9] Fraser D (1957). Hypophosphatasia. Am J Med.

[10] Kozlowski K, Sutcliffe J, Barylak A (1976). Hypophosphatasia. Review of 24 cases. Pediatr Radiol.

[11] Baumgartner-Sigl S, Haberlandt E, Mumm S (2007). Pyridoxine-responsive seizures as the first symptom of infantile hypophosphatasia caused by two novel missense mutations (c.677T>C, p.M226T; c.1112C>T, p.T371I) of the tissue-nonspecific alkaline phosphatase gene. Bone.

[12] Collmann H, Mornet E, Gattenlohner S (2009). Neurosurgical aspects of childhood hypophosphatasia. Childs Nerv Syst.

[13] Balasubramaniam S, Bowling F, Carpenter K (2010). Perinatal hypophosphatasia presenting as neonatal epileptic encephalopathy with abnormal neurotransmitter metabolism secondary to reduced co-factor pyridoxal-5′-phosphate availability. J Inherit Metab Dis.

[14] Silver MM, Vilos GA, Milne KJ (1988). Pulmonary hypoplasia in neonatal hypophosphatasia. Pediatr Pathol.

[15] Leung EC, Mhanni AA, Reed M (2013). Outcome of perinatal hypophosphatasia in Manitoba Mennonites: a retrospective cohort analysis. JIMD Rep.

[16] Nakamura-Utsunomiya A, Okada S, Hara K (2010). Clinical characteristics of perinatal lethal hypophosphatasia: a report of 6 cases. Clin Pediatr Endocrinol.

[17] Whyte MP, Rockman-Greenberg C, Ozono K (2016). Asfotase alfa treatment improves survival for perinatal and infantile hypophosphatasia. J Clin Endocrinol Metab.

[18] Mornet E, Yvard A, Taillandier A (2011). A molecular-based estimation of the prevalence of hypophosphatasia in the European population. Ann Hum Genet.

[19] Greenberg CR, Evans JA, McKendry-Smith S (1990). Infantile hypophosphatasia: localization within chromosome region 1p36.1-34 and prenatal diagnosis using linked DNA markers. Am J Hum Genet.

[20] Mehes K, Klujber L, Lassu G, Kajtar P (1972). Hypophosphatasia: screening and family investigations in an endogamous Hungarian village. Clin Genet.

[21] Rubecz I, Mehes K, Klujber L (1974). Hypophosphatasia: screening and family investigation. Clin Genet.

[22] Hofmann C, Girschick HJ, Mentrup B (2013). Clinical aspects of hypophosphatasia: an update. Clin Rev Bone Miner Metab.

[23] Bishop N, Munns CF, Ozono K (2016). Transformative therapy in hypophosphatasia. Arch Dis Child.

[24] Krakow D, Lachman RS, Rimoin DL (2009). Guidelines for the prenatal diagnosis of fetal skeletal dysplasias. Genet Med.

[25] Dighe M, Fligner C, Cheng E (2008). Fetal skeletal dysplasia: an approach to diagnosis with illustrative cases. Radiographics.

[26] Bonafe L, Cormier-Daire V, Hall C (2015). Nosology and classification of genetic skeletal disorders: 2015 revision. Am J Med Genet A.

[27] Schramm T, Gloning KP, Minderer S (2009). Prenatal sonographic diagnosis of skeletal dysplasias. Ultrasound Obstet Gynecol.

[28] Calder AD, Offiah AC (2015). Foetal radiography for suspected skeletal dysplasia: technique, normal appearances, diagnostic approach. Pediatr Radiol.

[29] Offiah AC (2015) Skeletal dysplasias: an overview. In: Allgrove J, Shaw NJ (eds) Calcium and bone disorders in children and adolescents, Vol. 28, 2nd edn, revised. Karger, Basel, Switzerland, pp 259–276

[30] Salomon LJ, Alfirevic Z, Bilardo CM (2013). ISUOG practice guidelines: performance of first-trimester fetal ultrasound scan. Ultrasound Obstet Gynecol.

[31] Antenatal care for uncomplicated pregnancies [clinical guideline CG62] (2016, Updated 2017) National Institute for Health and Care Excellence, London. Available via https://www.nice.org.uk/guidance/cg62 (accessed August 3, 2018)

[32] Krakow D, Williams J, Poehl M (2003). Use of three-dimensional ultrasound imaging in the diagnosis of prenatal-onset skeletal dysplasias. Ultrasound Obstet Gynecol.

[33] Victoria T, Epelman M, Bebbington M (2012). Low-dose fetal CT for evaluation of severe congenital skeletal anomalies: preliminary experience. Pediatr Radiol.

[34] Cassart M (2010). Suspected fetal skeletal malformations or bone diseases: how to explore. Pediatr Radiol.

[35] Guillerman RP (2011). Newer CT applications and their alternatives: what is appropriate in children?. Pediatr Radiol.

[36] Miyazaki O, Sawai H, Murotsuki J (2014). Nationwide radiation dose survey of computed tomography for fetal skeletal dysplasias. Pediatr Radiol.

[37] Victoria T, Epelman M, Coleman BG (2013). Low-dose fetal CT in the prenatal evaluation of skeletal dysplasias and other severe skeletal abnormalities. AJR Am J Roentgenol.

[38] Hall CM, Offiah A, Forzano F et al (2012) Diagnosis of fetal skeletal dysplasias. In: Fetal and perinatal skeletal dysplasias: an atlas of multimodality imaging. Radcliffe Publishing, London

[39] Sadro CT, Dubinsky TJ (2013). CT in pregnancy: risks and benefits. Appl Radiol.

[40] Imai R, Miyazaki O, Horiuchi T (2017). Ultra-low-dose fetal CT with model-based iterative reconstruction: a prospective pilot study. AJR Am J Roentgenol.

[41] Noel AE, Brown RN (2014). Advances in evaluating the fetal skeleton. Intl J Womens Health.

[42] Berceanu C, Gheonea IA, Vladareanu S (2017). Ultrasound and MRI comprehensive approach in prenatal diagnosis of fetal osteochondrodysplasias. Cases series. Med Ultrason.

[43] Nemec U, Nemec SF, Krakow D (2011). The skeleton and musculature on foetal MRI. Insights Imaging.

[44] Robinson AJ, Blaser S, Vladimirov A (2015). Foetal "black bone" MRI: utility in assessment of the foetal spine. Br J Radiol.

[45] Offiah AC, Hall CM (2003) Radiological diagnosis of the constitutional disorders of bone. As easy as A, B, C? Pediatr Radiol 33:153–161 10.1007/s00247-002-0855-8 12612812

[46] Beck C, Morbach H, Wirth C (2011). Whole-body MRI in the childhood form of hypophosphatasia. Rheumatol Int.

[47] Kritsaneepaiboon S, Jaruratanasirikul S, Dissaneevate S (2006). Clinics in diagnostic imaging (112). Perinatal lethal hypophosphatasia (PLH). Singap Med J.

[48] Matsushita M, Kitoh H, Michigami T (2014). Benign prenatal hypophosphatasia: a treatable disease not to be missed. Pediatr Radiol.

[49] Comstock C, Bronsteen R, Lee W, Vettraino I (2005). Mild hypophosphatasia in utero: bent bones in a family with dental disease. J Ultrasound Med.

[50] Pauli RM, Modaff P, Sipes SL, Whyte MP (1999). Mild hypophosphatasia mimicking severe osteogenesis imperfecta in utero: bent but not broken. Am J Med Genet.

[51] Gortzak-Uzan L, Sheiner E, Gohar J (2000) Prenatal diagnosis of congenital hypophosphatasia in a consanguineous Bedouin couple. A case report. J Reprod Med 45:588–590 10948473

[52] Moore CA, Curry CJ, Henthorn PS (1999). Mild autosomal dominant hypophosphatasia: in utero presentation in two families. Am J Med Genet.

[53] Souka AP, Raymond FL, Mornet E (2002). Hypophosphatasia associated with increased nuchal translucency: a report of two affected pregnancies. Ultrasound Obstet Gynecol.

[54] Tongsong T, Pongsatha S (2000). Early prenatal sonographic diagnosis of congenital hypophosphatasia. Ultrasound Obstet Gynecol.

[55] Sergi C, Mornet E, Troeger J, Voigtlaender T (2001). Perinatal hypophosphatasia: radiology, pathology and molecular biology studies in a family harboring a splicing mutation (648+1A) and a novel missense mutation (N400S) in the tissue-nonspecific alkaline phosphatase (TNSALP) gene. Am J Med Genet.

[56] Zankl A, Mornet E, Wong S (2008). Specific ultrasonographic features of perinatal lethal hypophosphatasia. Am J Med Genet A.

[57] Guguloth A, Aswani Y, Anandpara KM (2016). Prenatal diagnosis of hypophosphatasia congenita using ultrasonography. Ultrasonography.

[58] Shohat M, Rimoin DL, Gruber HE, Lachman RS (1991). Perinatal lethal hypophosphatasia; clinical, radiologic and morphologic findings. Pediatr Radiol.

[59] Arundel P, Offiah A, Bishop NJ (2011). Evolution of the radiographic appearance of the metaphyses over the first year of life in type V osteogenesis imperfecta: clues to pathogenesis. J Bone Miner Res.

[60] Calder AD (2015). Radiology of osteogenesis imperfecta, rickets and other bony fragility states. Endocr Dev.

[61] Wiebe S, Suchet I, Lemire EG (2007) Radiographic and prenatal ultrasound features of perinatal lethal hypophosphatasia - differentiation from osteogenesis imperfecta type II. S Afr J Radiol 11:32–35

[62] Oestreich AE, Bofinger MK (1989). Prominent transverse (Bowdler) bone spurs as a diagnostic clue in a case of neonatal hypophosphatasia without metaphyseal irregularity. Pediatr Radiol.

[63] Sinico M, Levaillant JM, Vergnaud A (2007). Specific osseous spurs in a lethal form of hypophosphatasia correlated with 3D prenatal ultrasonographic images. Prenat Diagn.

[64] Unger S, Mornet E, Mundlos S (2002). Severe cleidocranial dysplasia can mimic hypophosphatasia. Eur J Pediatr.

[65] Oestreich AE (2016). Bowdler spur also found in camptomelic dysplasia. Pediatr Radiol.

[66] Wenkert D, McAlister WH, Coburn SP (2011). Hypophosphatasia: nonlethal disease despite skeletal presentation in utero (17 new cases and literature review). J Bone Miner Res.

[67] Milks KS, Hill LM, Hosseinzadeh K (2016). Evaluating skeletal dysplasias on prenatal ultrasound: an emphasis on predicting lethality. Pediatr Radiol.

[68] Barros CA, Rezende Gde C, Araujo Junior E et al (2016) Prediction of lethal pulmonary hypoplasia by means fetal lung volume in skeletal dysplasias: a three-dimensional ultrasound assessment. J Matern Fetal Neonatal Med 29:1725–1730 10.3109/14767058.2015.1064887 26135769

[69] Backstrom MC, Kuusela AL, Maki R (1996). Metabolic bone disease of prematurity. Ann Med.

[70] Samson GR (2005). Skeletal dysplasias with osteopenia in the newborn: the value of alkaline phosphatase. J Matern Fetal Neonatal Med.

[71] Krakow D, Alanay Y, Rimoin LP (2008). Evaluation of prenatal-onset osteochondrodysplasias by ultrasonography: a retrospective and prospective analysis. Am J Med Genet A.

[72] Bulas DI, Stern HJ, Rosenbaum KN (1994). Variable prenatal appearance of osteogenesis imperfecta. J Ultrasound Med.

[73] Wu Q, Wang W, Cao L (2015). Diagnosis of fetal osteogenesis imperfecta by multidisciplinary assessment: a retrospective study of 10 cases. Fetal Pediatr Pathol.

[74] Sillence DO, Senn A, Danks DM (1979). Genetic heterogeneity in osteogenesis imperfecta. J Med Genet.

[75] Mornet E, Nunes ME, Pagon RA, Adam MP, Ardinger HH (2016). Hypophosphatasia. GeneReviews.

[76] Cordone M, Lituania M, Zampatti C (1989). In utero ultrasonographic features of campomelic dysplasia. Prenat Diagn.

[77] Mundlos S (1999). Cleidocranial dysplasia: clinical and molecular genetics. J Med Genet.

[78] Renaud A, Aucourt J, Weill J (2013). Radiographic features of osteogenesis imperfecta. Insights Imaging.

[79] Kim OH, Jin DK, Kosaki K (2013). Osteogenesis imperfecta type V: clinical and radiographic manifestations in mutation confirmed patients. Am J Med Genet A.

[80] Stewart PA, Wallerstein R, Moran E, Lee MJ (2000). Early prenatal ultrasound diagnosis of cleidocranial dysplasia. Ultrasound Obstet Gynecol.

[81] Hove HD, Hermann NV, Jorgensen C (2008). An echo-poor spine at 13 weeks: an early sign of cleidocranial dysplasia. Fetal Diagn Ther.

[82] Hermann NV, Hove HD, Jorgensen C (2009). Prenatal 3D ultrasound diagnostics in cleidocranial dysplasia. Fetal Diagn Ther.

[83] Morava E, Karteszi J, Weisenbach J (2002). Cleidocranial dysplasia with decreased bone density and biochemical findings of hypophosphatasia. Eur J Pediatr.

[84] El-Gharbawy AH, Peeden JN, Lachman RS (2010). Severe cleidocranial dysplasia and hypophosphatasia in a child with microdeletion of the C-terminal region of RUNX2. Am J Med Genet A.

[85] Benacerraf BR, Lister JE, DuPonte BL (1988) First-trimester diagnosis of fetal abnormalities. A report of three cases. J Reprod Med 33:777–780 3050079

[86] Suzumori N, Mornet E, Mizutani E (2011). Prenatal diagnosis of familial lethal hypophosphatasia using imaging, blood enzyme levels, chorionic villus sampling and archived fetal tissue. J Obstet Gynaecol Res.

[87] Takahashi Y, Sawai H, Murotsuki J (2017). Parental serum alkaline phosphatase activity as an auxiliary tool for prenatal diagnosis of hypophosphatasia. Prenat Diagn.

[88] Brock DJ, Barron L (1991). First-trimester prenatal diagnosis of hypophosphatasia: experience with 16 cases. Prenat Diagn.

[89] Mornet E, Muller F, Ngo S (1999). Correlation of alkaline phosphatase (ALP) determination and analysis of the tissue non-specific ALP gene in prenatal diagnosis of severe hypophosphatasia. Prenat Diagn.

[90] Muller F, Oury JF, Bussiere P (1991). First-trimester diagnosis of hypophosphatasia. Importance of gestational age and purity of CV samples. Prenat Diagn.

[91] Taillandier A, Domingues C, De Cazanove C (2015). Molecular diagnosis of hypophosphatasia and differential diagnosis by targeted Next Generation Sequencing. Mol Genet Metab.

[92] Kishnani PS, Rush ET, Arundel P (2017). Monitoring guidance for patients with hypophosphatasia treated with asfotase alfa. Mol Genet Metab.

[93] Forlenza GP, Calhoun A, Beckman KB (2015). Next generation sequencing in endocrine practice. Mol Genet Metab.

[94] Simon-Bouy B, Taillandier A, Fauvert D (2008). Hypophosphatasia: molecular testing of 19 prenatal cases and discussion about genetic counseling. Prenat Diagn.

